# Effects of magnesium-modified biochar on soil organic carbon mineralization in citrus orchard

**DOI:** 10.3389/fmicb.2023.1109272

**Published:** 2023-01-27

**Authors:** Lening Hu, Rui Huang, Liming Zhou, Rui Qin, Xunyang He, Hua Deng, Ke Li

**Affiliations:** ^1^Key Laboratory of Ecology of Rare and Endangered Species and Environmental Protection, Guangxi Normal University, Ministry of Education, Guilin, China; ^2^College of Environment and Resources, Guangxi Normal University, Guilin, China; ^3^Key Laboratory of Geospatial Technology for Middle and Lower Yellow River Regions, Henan University, Ministry of Education, Kaifeng, China; ^4^CAS Key Laboratory of Agro-ecological Processes in Subtropical Region, Institute of Subtropical Agriculture, Changsha, China; ^5^College of Civil Engineering and Architecture, Guilin University of Technology, Guilin, China

**Keywords:** Mg-modified citrus peel biochar, mineralization, organic carbon fraction, enzyme activity, soil physical and chemical properties

## Abstract

In order to investigate the carbon sequestration potential of biochar on soil, citrus orchard soils with a forest age of 5 years was taken as the research object, citrus peel biochar (OBC) and magnesium-modified citrus peel biochar (OBC-mg) were selected as additive materials, and organic carbon mineralization experiments were carried out in citrus orchard soil. OBC and OBC-Mg were applied to citrus orchard soils at four application rates (0, 1, 2, and 4%), and incubated at a constant temperature for 100 days. Compared with CK, the cumulative mineralization of soil organic carbon decreased by 5.11% with 1% OBC and 2.14% with 1% OBC-Mg. The application of OBC and OBC-Mg significantly increased the content of soil organic carbon fraction, while the content of soil organic carbon fraction was higher in OBC-Mg treated soil than in OBC treated soil. Meanwhile, the cumulative mineralization of soil organic carbon was significantly and positively correlated with the activities of soil catalase, urease and sucrase. The enzyme activities increased with the cumulative mineralization of organic carbon, and the enzyme activities of the OBC-Mg treated soil were significantly higher than those of the OBC treated soil. The results indicated that the OBC-Mg treatment inhibited the organic carbon mineralization in citrus orchard soils and was more favorable to the increase of soil organic carbon fraction. The Mg-modified approach improved the carbon sequestration potential of biochar for citrus orchard soils and provided favorable support for the theory of soil carbon sink in orchards.

## 1. Introduction

In recent years, the excessive emission of greenhouse gas has led to a series of serious environmental problems and posed a great threat to the survival of human beings. China is a large agricultural country, and agricultural soil is an important source of greenhouse gas emissions (Stefaner et al., 2021). Carbon dioxide is one of the most important greenhouse gases (Perez-Quezada et al., 2021). Scientists estimate that by 2050, atmospheric CO_2_ levels will reach 550 mg/l (Du et al., 2022). Carbon sequestration through soil to achieve emission reduction is one of the important directions of environmental research today. Soil organic carbon (SOC) is one of the most important indicators of soil quality (Masto et al., 2013a). Soil organic carbon affects the global carbon cycle process by influencing the release or sequestration of atmospheric CO_2_ from soil. Soil organic carbon mineralization is an important process for the conversion of carbon to CO_2_ in soil (Gan et al., 2020), therefore it is important to study the effect of soil organic carbon mineralization on the soil carbon cycle.

Previously, many materials have been used in the study of agricultural soil, and studies have shown that the application of dolomite and different biochar can reduce soil greenhouse gas emissions (Oo et al., 2018). The application of rice straw biochar at high CO_2_ concentration and air temperature can suppress soil NO_2_ emissions (Sun et al., 2018), which indicates that biochar has a better impact on reducing greenhouse gas emissions from agricultural soil (Korai et al., 2018; Wu et al., 2018). Biochar is a solid material with high carbon content prepared by high-temperature pyrolysis of biomass under oxygen-limited or oxygen-isolated conditions (Chen, W. et al., 2019). The effects of biochar application in soil have been extensively studied (Kuo et al., 2020; Rubin et al., 2020). Studies have shown that the application of biochar to soil reduces greenhouse gas emissions; and has a positive impact in terms of soil moisture retention, soil nitrogen and phosphorus retention (Bashir et al., 2020; Lu et al., 2020). At the same time, the application of biochar to soil can provide nutrients and habitat for microorganisms, thus directly or indirectly affecting the function and composition of microbial communities (Abhishek et al., 2022). In addition, the application of biochar increases the microbial pool of carbon and nitrogen in the soil and provides organic substrate for soil enzymes, thus changing the enzyme activity (Ullah et al., 2020). Therefore, applying biochar to the soil is important to improve soil nutrient dynamics and maintain the soil organic carbon pool.

Modification of biochar can improve its properties and enhance the stability of biochar (Liu et al., 2021). Mg is used for biochar modification because of its abundant reserves, non-toxicity, and high affinity for anions (Jiao et al., 2022), Mg is often used to make modified biochar. Yin et al. (2018) modified biochar with Mg and used it to treat PO_4_^3−⋅^ and NO_3_^−⋅^ contaminated eutrophic water bodies, and the results showed that Mg-modified biochar can improve water quality. The results of Wu et al. (2019) showed that application of MgO modified biochar to saline soil could increase soil effective phosphorus content and crop yield. Shan et al. (2022) applied Mg-modified peanut shell biochar to soil and effectively increased soil pH and reduced soil bioeffective state Cd content. Khan et al. (2022) even showed that Mg-modified biochar could slow down soil greenhouse gas emissions. Soil calcium and magnesium deficiencies are common in citrus soils (Chen, H. et al., 2019), and applying Mg-modified biochar to citrus soils can effectively improve the soil Mg deficiency. At present, the application of Mg-modified biochar to citrus soils has not been extensively studied, so the study of the effect of Mg-modified biochar on organic carbon mineralization in citrus soils is of great value.

Guangxi is the province with the largest citrus planting area and yield of citrus in China (Xie et al., 2021; Qiao et al., 2021). Bad and waste fruits as well as peel residues from citrus orchard cause a lot of wasted resources, and burning citrus peels into carbon applied to soil may be an important way to make efficient and reasonable utilization of waste *in situ*. Therefore, in this study, citrus peel biochar was modified to prepare Mg-modified citrus peel biochar, and citrus peel biochar and Mg-modified citrus peel biochar were applied to citrus orchard soils at different proportions. The effects on soil organic carbon mineralization, organic carbon and its active components in citrus orchard soils were investigated through a 100-day incubation experiment in a constant temperature incubation room. The effects and mechanisms of adding biochar on soil organic carbon mineralization were explored through the characteristics of changes in various indicators under different treatments.

## 2. Materials and methods

### 2.1. Experimental materials

The test soil was obtained in December 2021 from the core demonstration base of citrus cultivation at the Encounter Dragon River, Yangshuo County, Guilin City, Guangxi Zhuang Autonomous Region (24°48′17″N, 110°22′16″E). The area has a subtropical monsoon climate with an average annual temperature of 19.1°C and an average annual rainfall of 1887.6 mm. The soil type is red soil. Soil samples were collected from 0 to 20 cm surface layer of citrus orchards according to the principle of random multi-point mixing. Soil samples were removed from plant and animal residues and gravels, naturally dried and passed through 2 mm sieve for subsequent experiments. The basic physical and chemical properties of the test soils are shown in Table 1.

**Table 1:** Basic physical and chemical properties of the test soil.

pH	Ec (S m^−1^)	AP (mg kg^−1^)	AK (mg kg^−1^)	SOC (g kg^−1^)	Mechanical composition (%)	
4.36 ± 0.01	53.8 ± 2.07	27.13 ± 0.56	38.24 ± 1.21	6.78 ± 0.27	Sand (0.2–0.02 mm)	25.6
					Silt (0.02–0.002 mm)	47.1
					Clay (<0.002 mm)	27.3

Mean ± standard deviation of each index of the test soil (*n* = 3). EC, conductivity; AP, available phosphorus; AK, available potassium; SOC, soil organic carbon. The values followed by the same superscript letters within a row are not significantly different (*p* < = 0.05) between relevant treatments.

Citrus peels and soil were taken from the same citrus orchard. The citrus peels were dried at 70°C for 24 h, then crushed with a crusher and passed through a 60 mesh screen, and carbonized at 500°C for 2 h under oxygen limitation to prepare citrus peel biochar (OBC). The sieved citrus peel powder was mixed with 1 mol/l MgCl_2_–6H_2_O solution at a solid–liquid ratio of 1:10 for 24 h (That is, every 100 g of citrus peel powder mixed with 1 l of MgCl_2_–6H_2_O solution). The dried powder was filtered through a filter and dried at 60°C for 24 h. The Mg-modified citrus peel biochar (OBC-Mg) was prepared by limiting oxygen carbonization at 500°C for 2 h. The basic physicochemical properties of OBC and OBC-Mg are shown in Table 2.

**Table 2:** Basic properties and elemental contents of two biochar.

	pH	Productivity (%)	C/N	C/H	Elemental contents (%)			
					C	H	N	S
OBC	10.04	38.13	36.72	114.59	63.77	0.556	1.74	0.009
OBC-Mg	10.19	46.26	35.87	73.07	23.81	0.326	0.66	0.029

### 2.2. Experimental design

There were seven treatments with three replications for each treatment, and the experiment was set up with four application rates, i.e., 0% (CK), 1, 2, and 4% of soil mass of the applied material, as shown in Table 3.

**Table 3:** Experiment design.

Number	1	2	3	4	5	6	7
Treatment	CK	1%OBC	2%OBC	4%OBC	1%OBC-Mg	2%OBC-Mg	4%OBC-Mg

Culture test: 1,000 g of soil was placed in 2 l polyethylene bottles. Citrus peel biochar and magnesium modified citrus peel biochar were applied according to the experimental design (Table 3). After the soil was mixed well with the materials, deionized water was added to keep the field water holding capacity at about 60%. The polyethylene bottles were incubated in a constant temperature incubator at 25°C for 100 days and soil samples were collected for analysis on days 1, 3, 5, 10, 15, 20, 30, 40, 60, 80, and 100, respectively. Another batch of soil samples of the same treatment was set up and incubated under the same conditions with a soil weight of 50 g. A 10 ml beaker containing 0.1 mol L^–1^ sodium hydroxide solution was placed in a polyethylene bottle and analyzed for soil organic carbon mineralization on days 1, 3, 5, 10, 15, 20, 30, 40, 60, 80, and 100 (Xu et al., 2019).

### 2.3. Measurement method

The pH was determined by potentiometric method (water–soil ratio 2.5:1; Zhang et al., 2021). Available phosphorus was determined by sodium bicarbonate extraction-molybdenum antimony anti-spectrophotometric method (Wang, H. et al., 2021). Available potassium was determined by 1 mol/l ammonium acetate extraction-flame photometric method (Rong et al., 2021). Soil cation exchange capacity (CEC) was determined by the barium chloride-sulfuric acid forced exchange method (Zhang et al., 2010). Soil organic carbon (SOC) was determined by the potassium dichromate oxidation-spectrophotometric method (Hu et al., 2020). Soil dissolved organic carbon (DOC) content was determined by TOC total organic carbon analyzer (Huang et al., 2018). Soil microbial biomass carbon (MBC) was determined by fumigation method (Gao et al., 2017).Soil readily organic carbon (ROC) was determined by 333 mmol/l potassium permanganate oxidation method (Jien et al., 2018). Soil catalase was determined by the potassium permanganate titration method (Zhao et al., 2020), soil urease activity was determined by the indophenol blue colorimetric method (Sarma et al., 2017), and soil sucrase activity was determined by the 3,5-dinitrosalicylic acid colorimetric method (Xu et al., 2019). Soil CO_2_ emissions were determined by the titrimetric method (Pei et al., 2017). The surface morphological characteristics of biochar were observed by FEI Inspect F5 field emission scanning electron microscope (SEM) at a magnification of 500–40,000 × and an acceleration voltage of 20 kV of the electron beam, and its composition was analyzed by mapping (Deng et al., 2022).

### 2.4. Calculation methods

(1) Soil organic carbon mineralization (in terms of CO_2_).

$$\begin{array}{l} {\mathrm{CO}}_{2}\left(\mathrm{mg}\cdot {\mathrm{kg}}^{-1}\right)=\\ \;\;\;\;\;\;\;\;\;\;\;\;\left\{\left[\left(V_{0}-V\right)\times c\times 0.022\times \left(22.4/44\right)\times 1000\right]\times 2\times 1000\right\}/m. \end{array}$$

(2) Soil organic carbon mineralization rate.
  Soil organic carbon mineralization rate (mg kg^−1^ d^−1^) = organic carbon mineralization/∆*t* (2).
(3) Cumulative soil organic carbon mineralization (total soil CO_2_ release from the beginning of cultivation to a certain time point).

$$\mathrm{Cumulative}\;\;\;\mathrm{mineralization}=\overset{n}{\underset{1}{\sum }}{\mathrm{CO}}_{2}.$$

(4) Mineralization fitting calculation.
  The first level kinetic equation was applied to fit the soil carbon mineralization under different treatments.

$$C_{t}=C_{0}\left(1-e^{-kt}\right)$$

where *V*_0_ is the volume of standard hydrochloric acid consumed during the blank titration, *V* is the volume of standard hydrochloric acid consumed during the sample titration, *c* is the concentration of standard hydrochloric acid, 0.022 is the molar mass of carbon dioxide (1/2CO_2_), *M* (1/2CO_2_) = 0.022 g mmol^−1^, 22.4 × 1000/44 is the number of milliliters per gram of CO_2_ in the standard state; △t is the incubation interval (*d*); *C_t_* is the cumulative mineralization at incubation time t (d), *C*_0_ is the potential mineralization of soil carbon, *C* (mg∙kg^−1^); *k* is the rate constant of soil carbon mineralization, d^−1^; *t* is the incubation time, day (d) (Hu et al., 2020).

### 2.5. Statistical analysis

Excel 2020 and SPSS 25.0 (IBM Corporation, United States) were used for statistical analysis of the data. One-way analysis of variance (ANOVA) was used to compare the differences between treatments, and the least significant difference (LSD) method was used to test the significance of differences at the level of significance (*p* < = 0.05). Figures were plotted by origin2022 (OriginLab Corporation, United States). The correlation heat map was plotted using the Correlation Plot App in origin 2022, with red representing positive correlations and blue representing negative correlations, and color shades representing the magnitude of correlation coefficients; the darker the color, the stronger the correlation.

## 3. Results

### 3.1. Characterization of biochar

**Figure 1:**
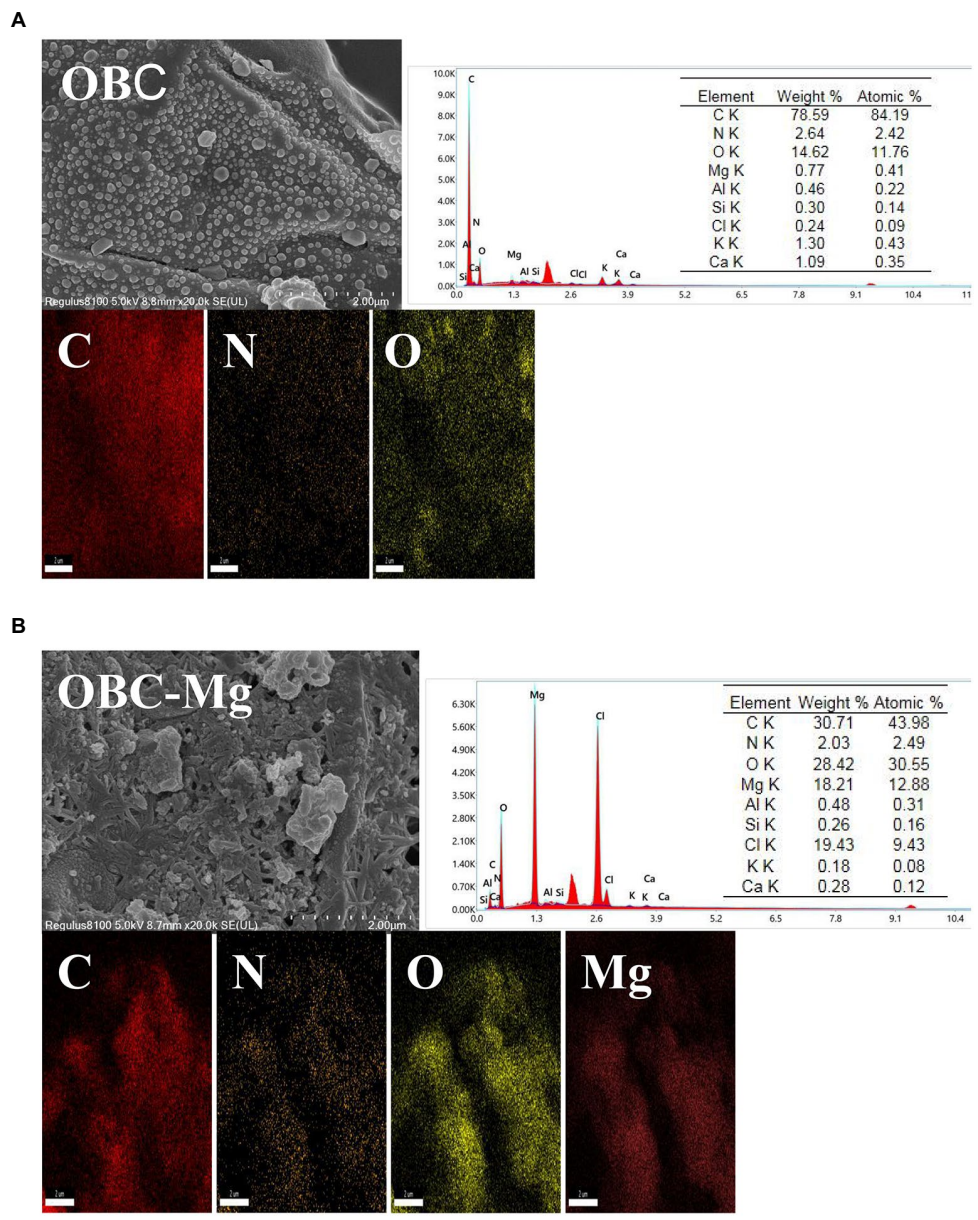
scanning electron microscopy–energy dispersive X-ray spectroscopy (SEM–EDS) analysis of **(A)** citrus peel biochar (OBC) and **(B)** magnesium-modified citrus peel biochar (OBC-Mg).

Figure 1 shows the SEM-EDS patterns of OBC and OBC-Mg. The morphological structures of OBC and OBC-Mg are significantly different. The surface structure of OBC in the figure presents a complete blocky structure with smooth surface and well-developed pore structure. The surface structure of OBC-Mg was broken, with increased specific surface area and irregular granular flocs on the surface. The rough flake morphology on the surface of OBC-Mg may be due to the formation of MgO from MgCl_2_–6H_2_O after intense dehydration in pyrolysis (Zhang et al., 2012; Li et al., 2017). The results of EDS analysis showed that that the elemental C content of the modified biochar surface decreased from 78.59 to 30.71% compared to OBC-Mg. The decrease in carbon content may be due to the absorption of Mg into the surface of carbon (Li et al., 2016). EDS analysis also showed that the content of Mg in OBC-Mg was significantly higher compared to OBC, which also indicated that the impregnation modification successfully loaded Mg. In addition, EDS analysis also revealed Al, Si, Cl, K, and *Ca.*

### 3.2. Effect of the OBC and OBC-Mg on soil physiochemical properties

#### 3.2.1. Impact of OBC and OBC-Mg on soil pH

**Figure 2:**
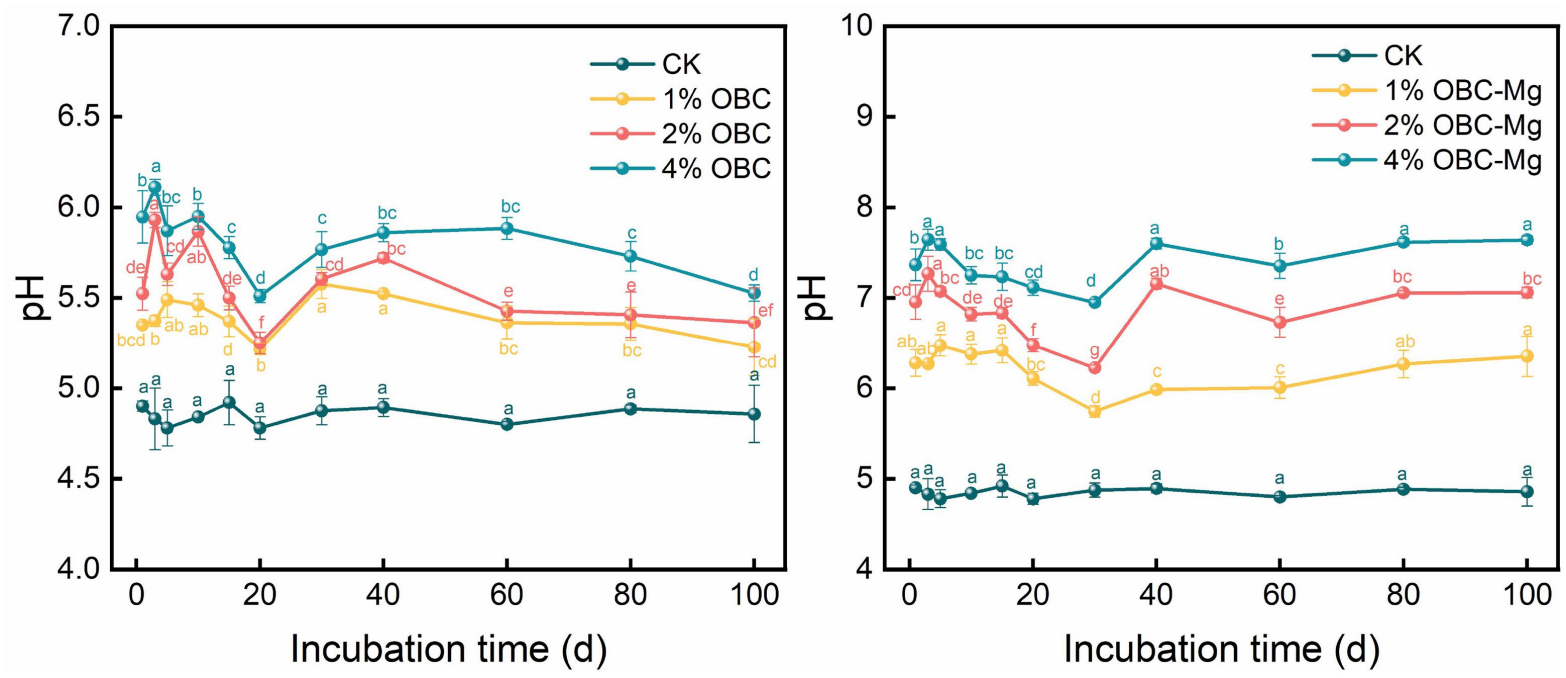
Changes in soil pH after the amendment of OBC and OBC-Mg.

The effects of OBC and OBC-Mg on soil pH are shown in Figure 2. The ANOVA/statistical indicators are shown in Supplementary Tables 1–3. The pH of CK without biochar application did not change significantly during the incubation period; the soil pH of OBC and OBC-Mg application increased significantly, and the soil pH of the same treatment increased significantly with the incubation time, and the improvement effect on soil acidity was shown as 4% > 2% > 1% > CK in both cases. After OBC application, the soil pH increased with the increase of application rate at the end of incubation compared with the control by 0.37, 0.51, and 0.67 units, respectively. After OBC-Mg application, soil pH increased by 1.50, 2.20, and 2.78 units at the end of the incubation compared to the CK. With the increase in the application rate, the difference between the different application rates was significant. The application of OBC-Mg was more effective in improving the acidity of the soil compared to other treatments.

#### 3.2.2. Impact of OBC and OBC-Mg on soil cation exchange capacity (CEC)

**Figure. 3:**
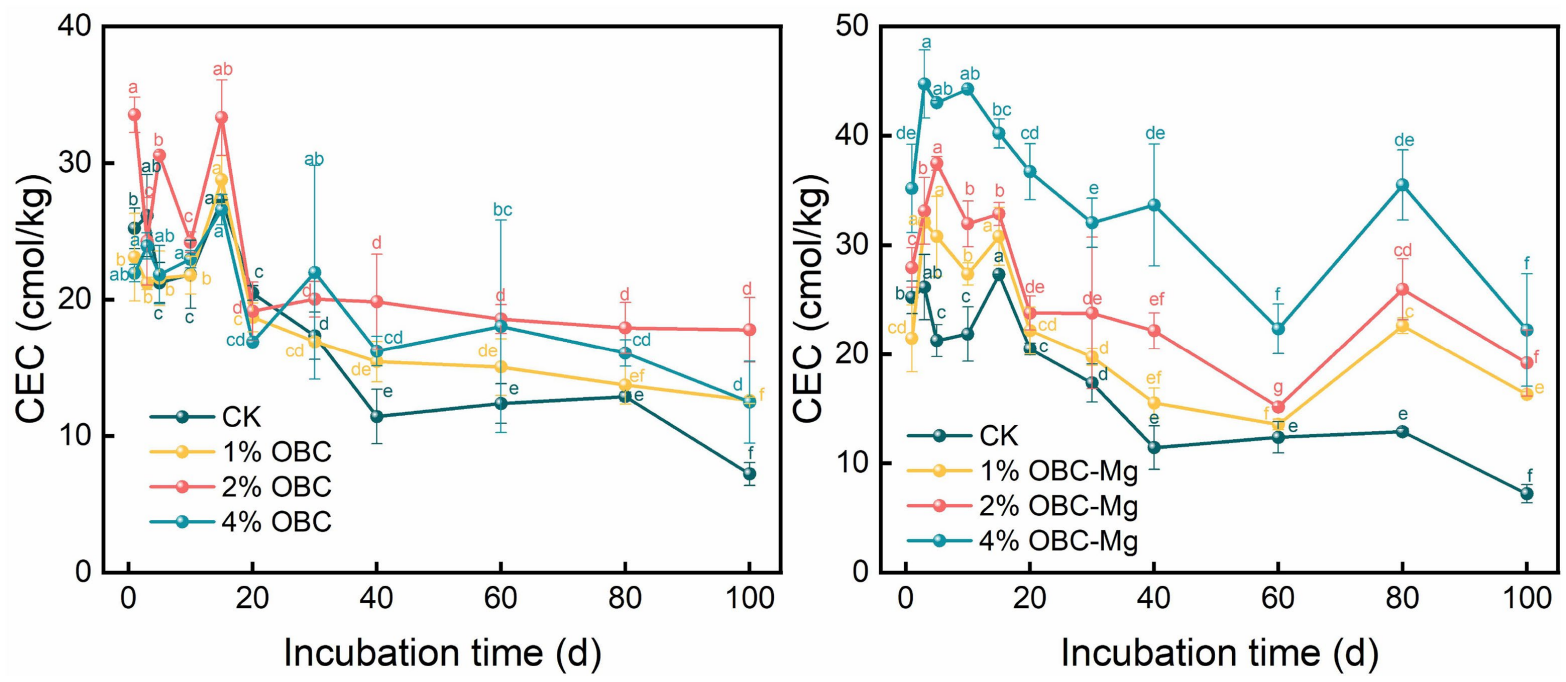
Changes in soil cation exchange capacity (CEC) after the amendment of OBC and OBC-Mg.

During the incubation period, the overall soil CEC showed a decreasing trend after the application of OBC and OBC-Mg with the increase of incubation time (Figure 3). Among them, CK soil had the highest CEC value at the 15th day of incubation and the lowest at the end of incubation; after applying OBC, 4% OBC increased soil CEC, 2% OBC and 1% OBC were partially slightly lower than CK at the 1st–30th days of incubation, and both were higher than CK after 30 days, and the difference of soil CEC of the same treatment with increasing incubation time was significant, and the effect on soil CEC was shown as 4% OBC > 2% OBC > 1% OBC. After OBC application, compared with CK at the end of incubation with the increase of the application rate, the soil CEC increased by 74.57, 146.49 and 72.88%, respectively. The application of OBC-Mg significantly increased the soil CEC, and the effect on CEC was 4% OBC-Mg > 2% OBC-Mg > 1% OBC-Mg. After the application of OBC-Mg, at the end of the incubation with the increase of the applied proportion compared to CK, the soil CEC increased by 125.74, 165.85, and 207.80%, respectively, and the difference between different applied proportions was significant. OBC-Mg was more effective in enhancing soil CEC compared to other treatments.

#### 3.2.3. Impact of OBC and OBC-Mg on soil available phosphorus (AP)

**Figure 4:**
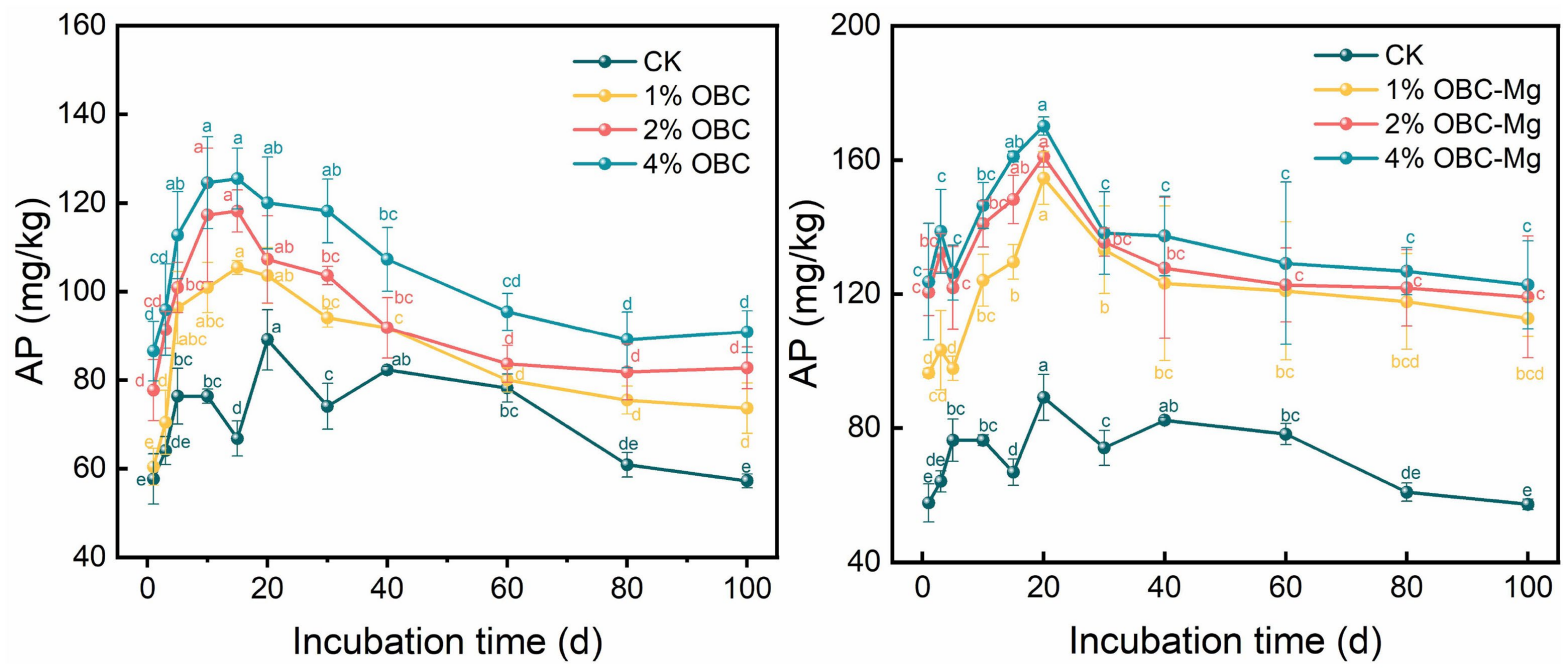
Changes in soil available phosphorus (AP) after the amendment of OBC and OBC-Mg.

The effects of OBC and OBC-Mg application on AP in citrus orchard soil are shown in Figure 4. After the application of OBC, the AP content of each treatment first increased, then decreased and gradually stabilized, reaching the highest level at the 15th day of incubation. At the end of the incubation, the AP content was higher than that of CK under different rates of OBC. The size of AP content in different rates of OBC application was 4% > 2% > 1% > CK. With the increase of application rate, the AP content increased by 28.62, 44.52 and 48.82% compared with CK. The trend of AP content in each treatment was similar to that of OBC, which first increased, then decreased and gradually stabilized, and reached the highest value at day 20 of incubation. The AP content of OBC-Mg applied at different rates was positively correlated with the application rate. Compared with CK, the AP content increased by 96.98, 108.11 and 114.47%, respectively. In a comprehensive comparison, OBC-Mg applied to citrus orchard soil increased the AP content better than OBC.

#### 3.2.4. Impact of OBC and OBC-Mg on soil available potassium (AK)

**Figure 5:**
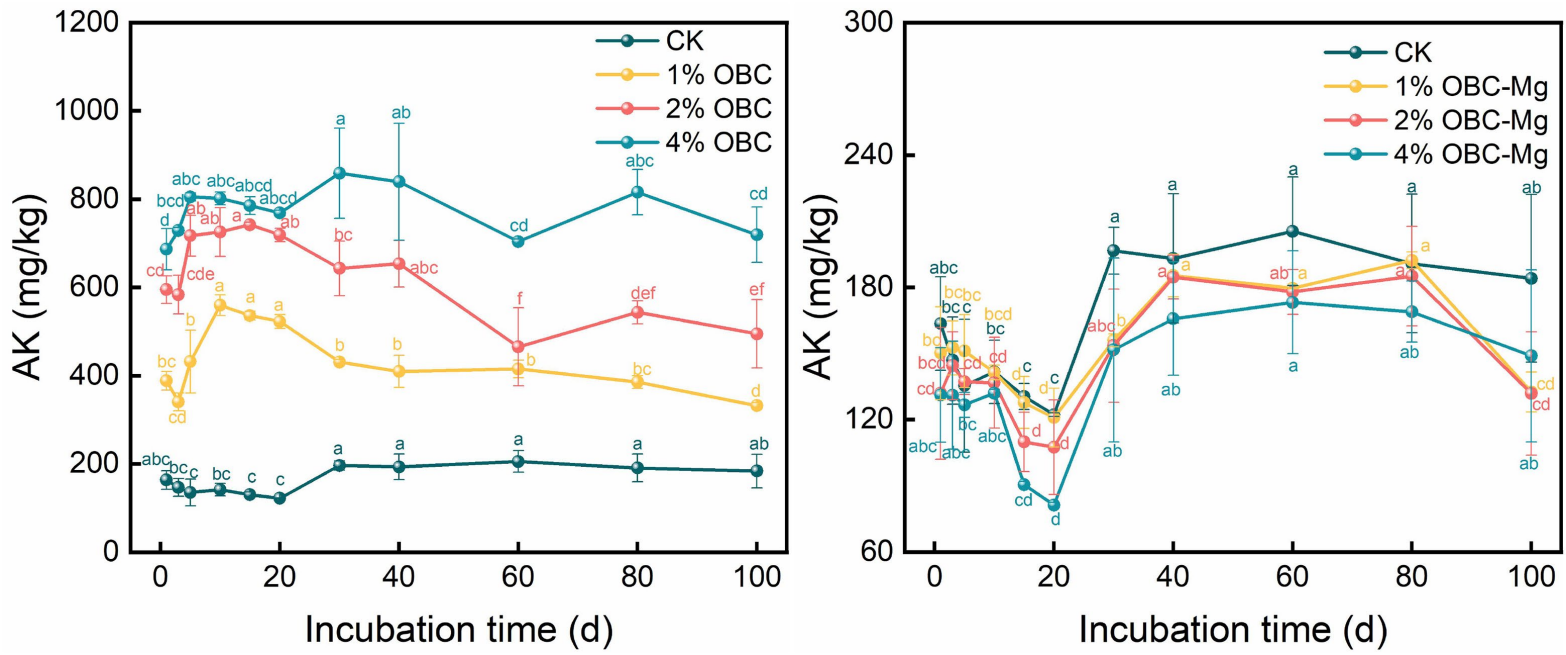
Changes in soil available potassium (AK) after the amendment of OBC and OBC-Mg.

The effects of OBC and OBC-Mg application on AK in citrus orchard soil are shown in Figure 5. After applying OBC-Mg to the soil, the AK content in the soil increased with the increase of the applied proportion. The AK content was 4% OBC > 2% OBC > 1% OBC > CK from the largest to the smallest. Compared with CK, at the end of the incubation, the AK content increased by 14.98, 18.61, and 19.12%, respectively, with the increase of the applied proportion. After applying different proportions of OBC-Mg to the soil, the AK content in the soil was CK > 1% OBC-Mg > 2% OBC-Mg > 4% OBC-Mg. Compared with CK, at the end of the incubation, the CK content decreased by 14.98, 18.61, and 19.12%, respectively, with the increase of the applied proportion.

### 3.3. Effect of the OBC and OBC-Mg on soil mineralization

#### 3.3.1. Impact of OBC and OBC-Mg on soil mineralization rate

**Figure 6:**
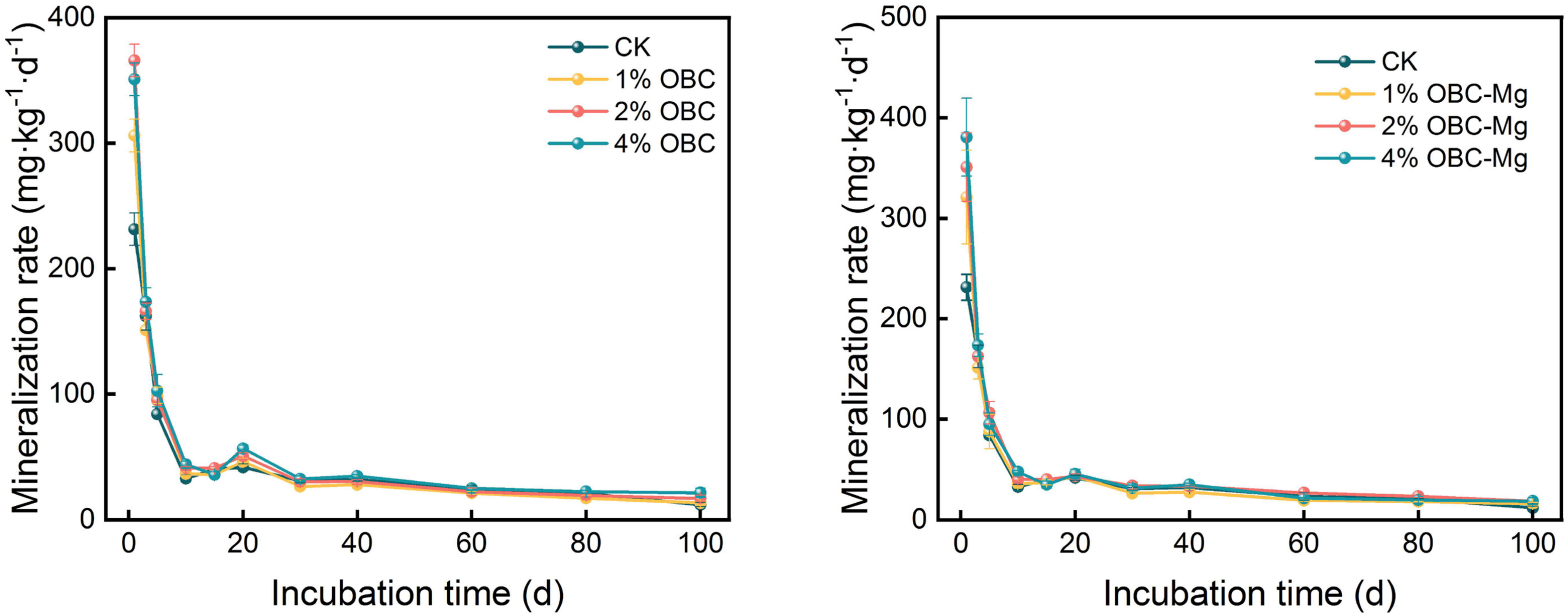
Changes in soil mineralization rate after the amendment of OBC and OBC-Mg.

As shown in Figure 6, the application of OBC and OBC-Mg to citrus orchard soil significantly affected the rate of soil organic carbon mineralization in citrus orchards. Throughout the incubation period, the soil organic carbon mineralization rates after applying different rates of OBC and OBC-Mg were higher than those of the CK. On the first day of incubation, the rates of soil organic carbon mineralization with 1, 2 and 4% OBC were 1.32, 1.58 and 1.52 times higher than those with CK, respectively. And the rates of soil organic carbon mineralization with 1, 2 and 4% OBC-Mg were 1.39, 1.52 and 1.65 times higher than those with CK. The soil mineralization rate decreased in all treatments from 0 to 10 days of incubation, and showed an increasing trend in all treatments from 10 to 20 days of incubation. The organic carbon mineralization rate decreased with increasing incubation time in all treatments after 20 days of incubation. At the end of incubation, compared with CK, soil organic carbon mineralization rates increased by 11.72, 41.21, and 78.45% with 1, 2, and 4% OBC applied; and increased by 33.45, 55.17, and 55.17% with 1, 2, and 4% OBC-Mg applied. In summary, all soil organic carbon mineralization rates decreased with increasing incubation time. The rates were at a high level in the early incubation period, decreased with increasing time, and stabilized in the late incubation period.

#### 3.3.2. Impact of OBC and OBC-Mg on soil cumulative mineralization

**Figure 7:**
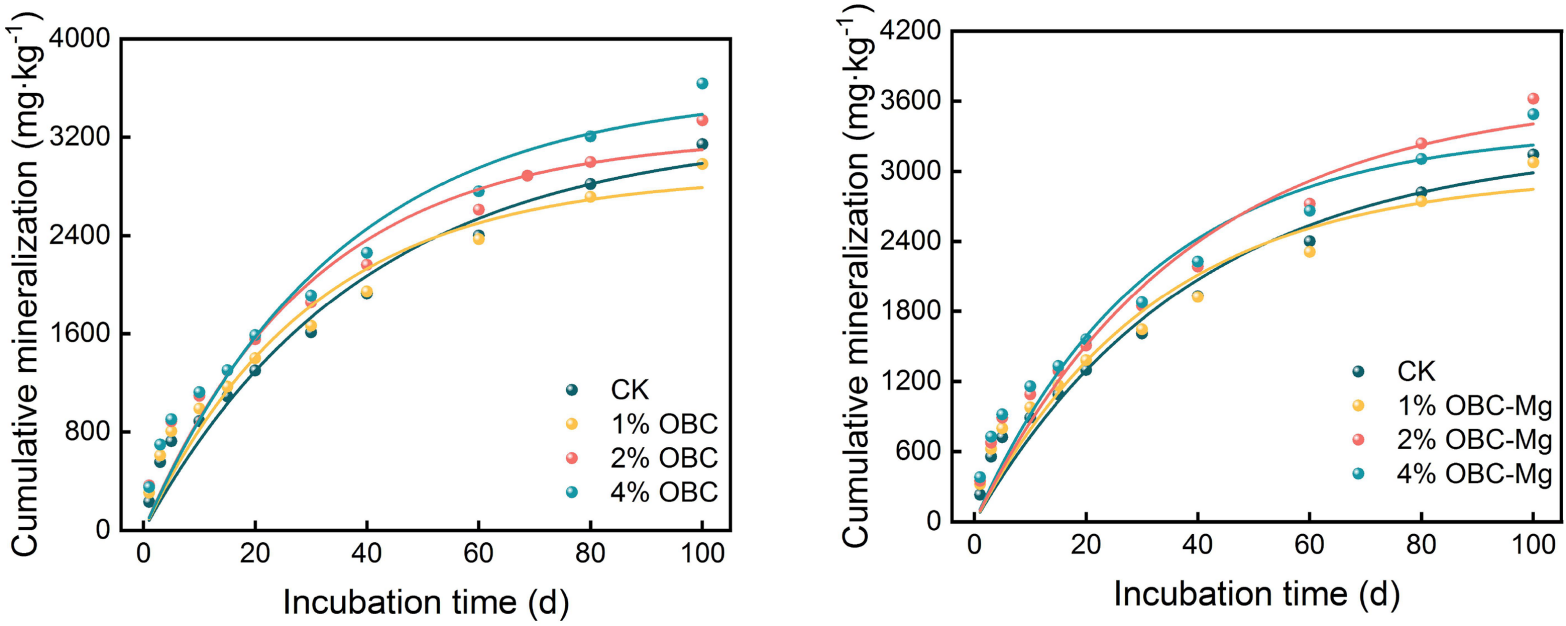
Changes in soil cumulative mineralization after the amendment of OBC and OBC-Mg.

As shown in Figure 7, the application of OBC and OBC-Mg significantly increased the cumulative soil organic carbon mineralization. The cumulative mineralization of soil organic carbon in CK was the lowest among the treatments from 0 to 40 days of incubation, and after 40 days of incubation, the cumulative mineralization of soil organic carbon in CK was higher than that in 1% OBC and 1% OBC-Mg. The cumulative mineralization of soil organic carbon in each treatment increased with the increase of incubation time. After the application of OBC, the cumulative mineralization of 1% OBC decreased by 5.11, 2% OBC and 4% OBC increased by 6.18 and 15.68%, respectively, compared with CK. The cumulative mineralization of soil organic carbon showed an overall increase of 4% OBC > 2% OBC > 1% OBC with the increase of the application ratio. At the end of incubation, the cumulative mineralization of 1% OBC-Mg decreased by 2.14, 2% OBC-Mg and 4% OBC-Mg increased by 15.20 and 10.93%, respectively. The cumulative mineralization showed 4% OBC-Mg > 2% OBC-Mg > 1% OBC-Mg at days 0–40 of incubation, and 2% OBC-Mg > 4% OBC-Mg > 1% OBC-Mg at days 40–100 of incubation. The application of 1% of two biochar to citrus orchard soils could slow down the emission of soil CO_2_ to some extent.

The dynamic changes between the cumulative mineralization of soil organic carbon and incubation days in citrus orchards after the application of OBC and OBC-Mg were fitted using the quasi-level kinetic equation *C*_t_ = *C*_0_(1–*e*^–kt^), and the model fitted well with a correlation coefficient *R*^2^ > 0.933 (Table 4). The results showed that after the application of OBC, the *C*_0_ of potential mineralization of organic carbon in 1% OBC and 2% OBC soils was lower than that of CK. The potential mineralization of organic carbon in 4% OBC soils was higher than that of CK. The organic carbon turnover rate constant k was significantly higher than that of CK, its *C*_0_ increased with the increase of the application ratio. After the application of OBC-Mg, the potential mineralization of organic carbon in 1% OBC-Mg soil *C*_0_ was lower than that of CK, the potential mineralization of organic carbon in 2% OBC-Mg and 4% OBC-Mg soil was higher than that of CK, the organic carbon turnover rate constant k was significantly higher than that of CK, its *C*_0_ increased with the increase of the application rate.

**Table 4:** Soil carbon mineralization kinetic parameters.

Treatment	Fitting parameters		
	C_0_/mg kg^−1^	k/d^−1^	*R* ^2^
CK	3,239 ± 257.40	0.026 ± 0.004	0.962
1% OBC	2,894 ± 208.92	0.033 ± 0.006	0.944
2% OBC	3,215 ± 240.54	0.033 ± 0.006	0.939
4% OBC	3,586 ± 294.67	0.029 ± 0.005	0.945
1% OBC-Mg	2,983 ± 248.38	0.031 ± 0.006	0.935
2% OBC-Mg	3,362 ± 274.01	0.032 ± 0.006	0.933
4% OBC-Mg	3,662 ± 330.20	0.027 ± 0.005	0.945

### 3.4. Effect of the OBC and OBC-Mg on soil active organic carbon components

#### 3.4.1. Impact of OBC and OBC-Mg on soil organic carbon

**Figure 8:**
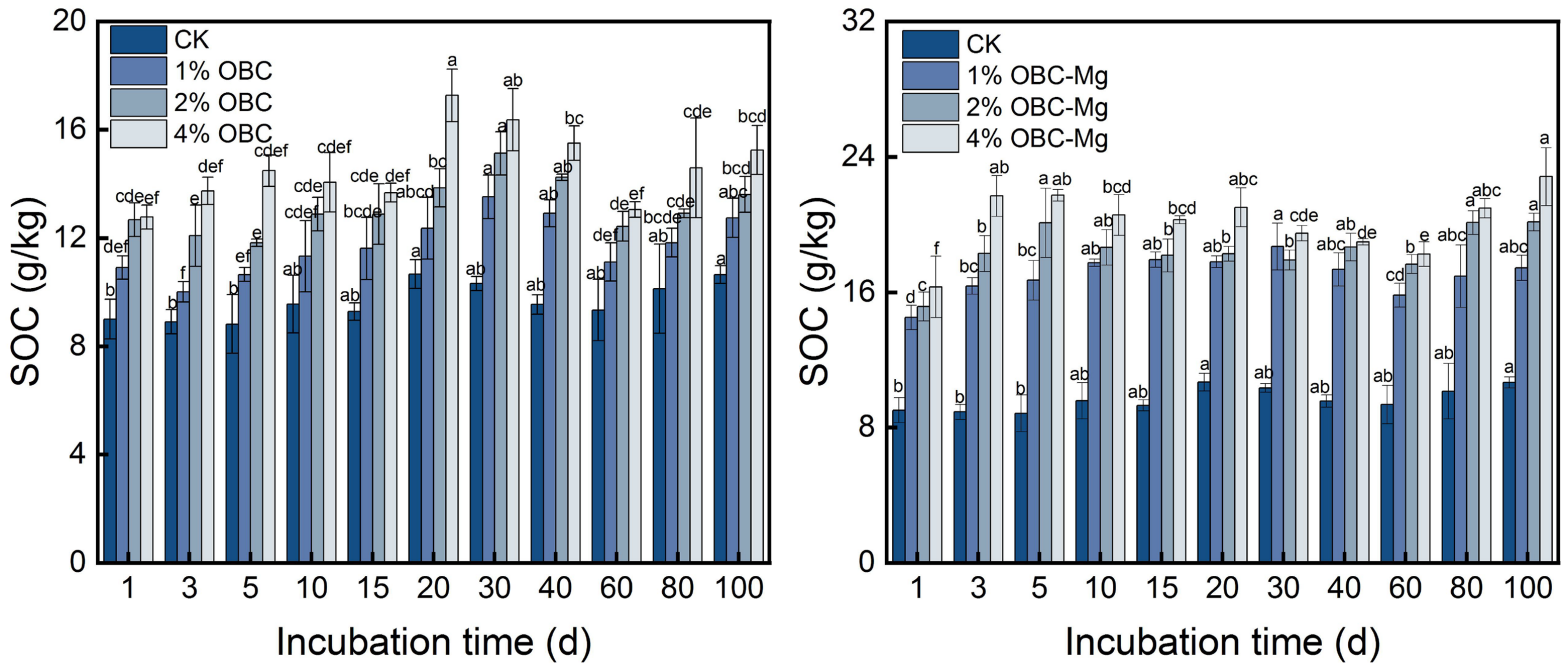
Changes in soil organic carbon (SOC) after the amendment of OBC and OBC-Mg.

Application of OBC and OBC-Mg to citrus orchard soil was able to significantly increase SOC content (Figure 8). The overall effect of OBC and OBC-Mg on the dynamics of soil SOC content showed 4% > 2% > 1% > CK, and the difference in the same application rate was not significant with increasing incubation time. Compared with CK, at the end of incubation, 1% OBC was 1.20 times, 2% OBC was 1.28 times and 4% OBC was 1.43 times of CK, 1% OBC-Mg was 1.64 times, 2% OBC-Mg was 1.89 times and 4% OBC-Mg was 2.14 times of CK. Compared with OBC, OBC-Mg applied to citrus orchard soil could better improve the SOC content in the soil.

#### 3.4.2. Impact of OBC and OBC-Mg on soil microbial biomass carbon

**Figure 9:**
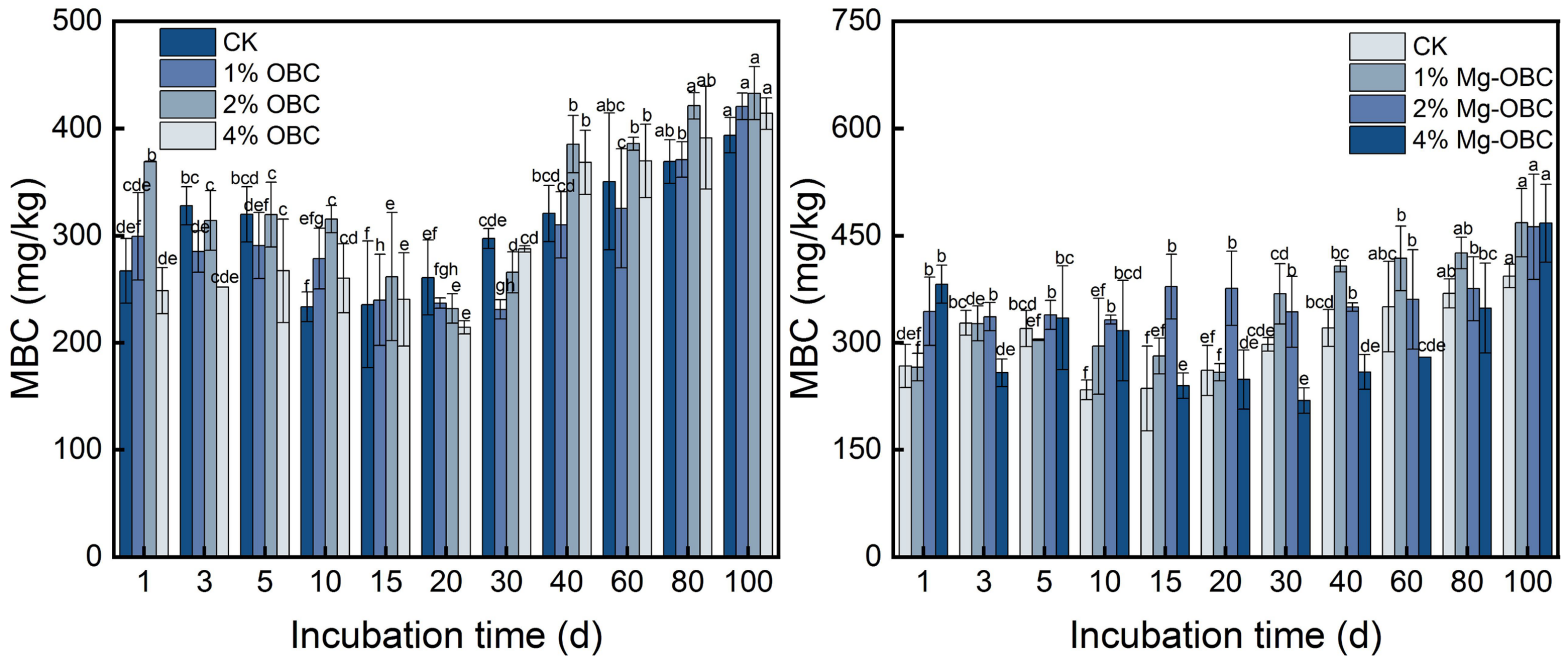
Changes in microbial biomass carbon (MBC) after the amendment of OBC and OBC-Mg.

As shown in Figure 9, the application of OBC and OBC-Mg to citrus orchard soil significantly changed the soil MBC content. After applying OBC to citrus orchard soils, soil MBC content gradually decreased from 0 to 20 days of incubation and gradually increased after 20 days, but some treatments still had less MBC than CK. At the end of incubation, 1% OBC increased by 6.77, 2% OBC increased by 9.92, and 4% OBC increased by 5.12% compared to CK. Overall, the application of 2% OBC could better increase soil MBC. After applying OBC-Mg to citrus orchard soil, soil MBC content changed repeatedly during the first 20 days of incubation and gradually increased from 30 to 100 days of incubation. At the end of incubation, the MBC content was greater than CK at all rates. At the end of incubation, 1% OBC-Mg increased by 18.95, 2% OBC-Mg increased by 17.50, and 4% OBC-Mg increased by 18.78% compared to CK.

#### 3.4.3. Impact of OBC and OBC-Mg on soil dissolved organic carbon

**Figure 10:**
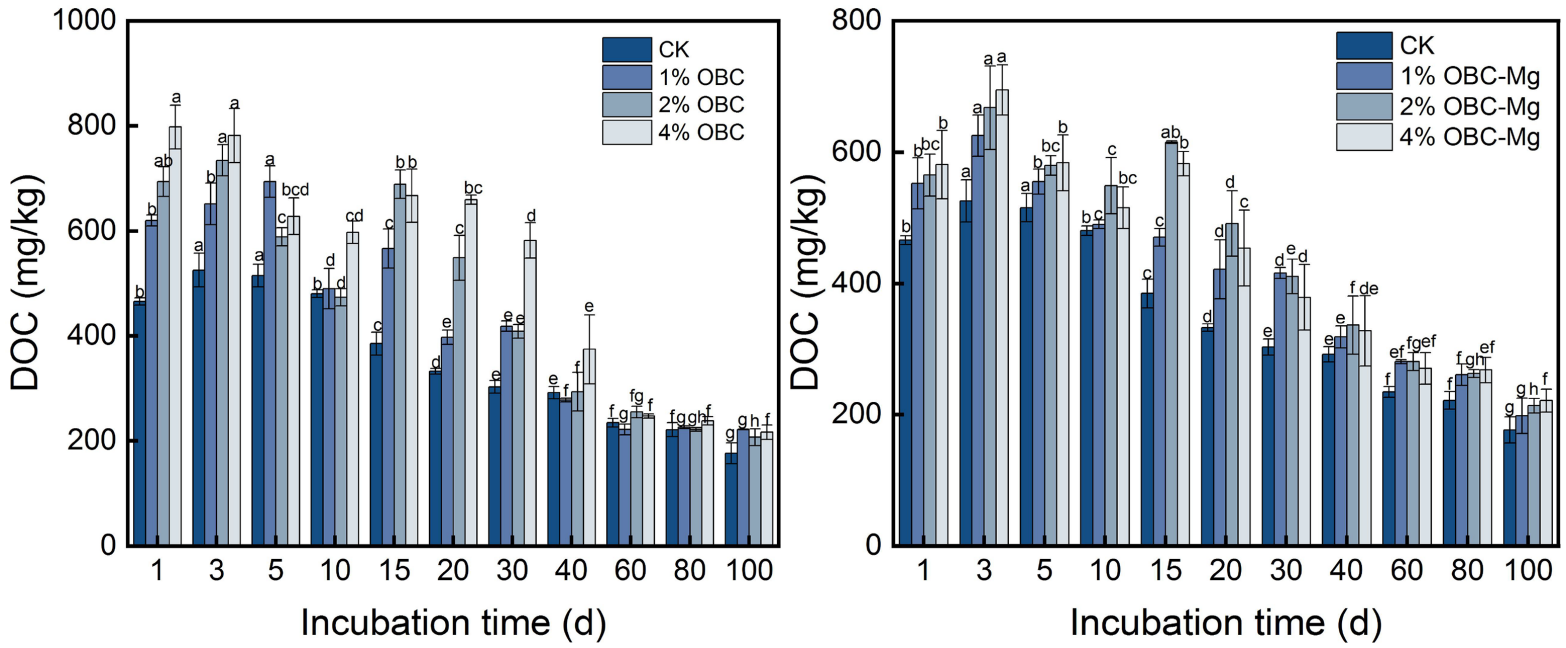
Changes in dissolved organic carbon (DOC) after the amendment of OBC and OBC-Mg.

As shown in Figure 10, the application of OBC and OBC-Mg to citrus orchard soil changed the DOC content in the soil. All treatments showed a decreasing trend with the increase of incubation time. After the application of OBC, each proportion of DOC showed a decreasing trend, but there were still some treatments with DOC less than CK, and at the end of incubation each applied proportion of DOC was greater than CK. Compared with CK, the DOC content increased by 25.67% (1% OBC), 17.26% (2% OBC), and 22.58% (4% OBC), respectively. After the application of OBC-Mg, DOC gradually increased in all ratios and at the end of the incubation, all treatments were greater than CK, and DOC content increased by 12.26% (1% OBC-Mg), 20.91% (2% OBC-Mg), and 25.28% (4% OBC-Mg), respectively.

#### 3.4.4. Impact of OBC and OBC-Mg on soil readily oxidized organic carbon

**Figure 11:**
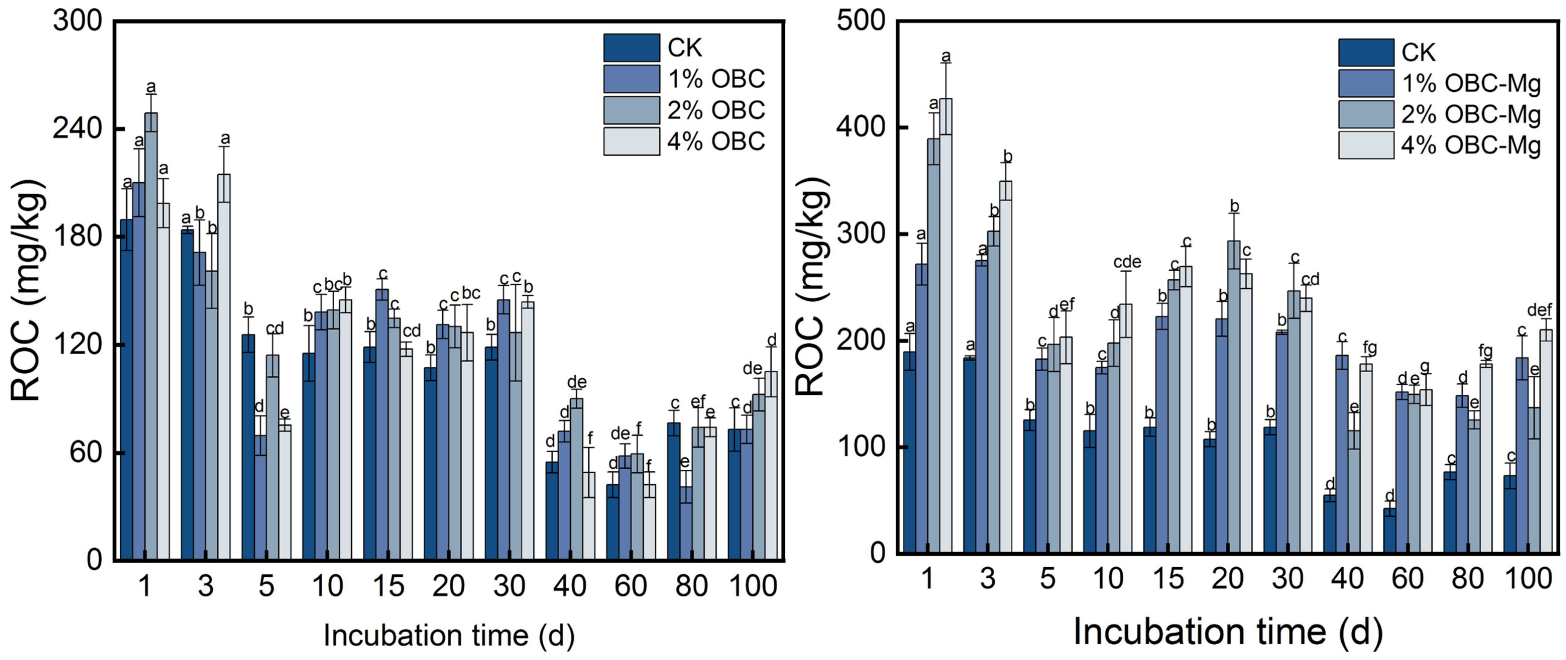
Changes in readily oxidized organic carbon (ROC) after the amendment of OBC and OBC-Mg.

As shown in Figure 11, the application of OBC and OBC-Mg to citrus orchard soil significantly changed the soil ROC content. After applying OBC to citrus orchard soils, the soil ROC content showed a decreasing trend throughout the incubation period, but some treatments still had less ROC than CK. At the end of incubation, the ROC content in 1% OBC soil was the same as CK, while 2% OBC increased by 26.56 and 4% OBC increased by 43.75% compared to CK. After applying OBC-Mg to citrus orchard soils, soil ROC content decreased on 0–5 days of incubation, gradually increased on 5–20 days, and increased after decreasing on 20–60 days. At the end of incubation, the ROC content of each treatment was greater than that of CK, 1% OBC-Mg increased by 151.56, 2% OBC-Mg increased by 87.50, and 4% OBC-Mg increased by 187.50% compared to CK.

### 3.5. Effect of the OBC and OBC-Mg on soil enzyme activity

#### 3.5.1. Impact of OBC and OBC-Mg on soil catalase activity

**Figure 12:**
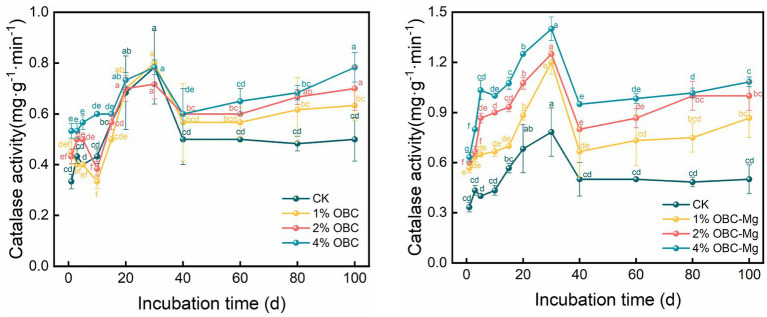
Changes in soil catalase activity after the amendment of OBC and OBC-Mg.

As shown in Figure 12, application of OBC and OBC-Mg to citrus orchard soils increased the activity of catalase in the soil. Compared with CK, after the application of OBC, the catalase activity in soil increased from 0 to 30 days of incubation, but some treatments still had less catalase activity than CK. Throughout the incubation period, the catalase activity first increased to reach the highest value at 30th day, then decreased and then stabilized. At the end of incubation, with the application ratio with an overall performance of 4% OBC > 2% OBC > 1% OBC > CK, it increased by 26.67, 40 and 56.67%, respectively, with the increase of applied ratio. After the application of OBC-Mg, the catalase activity in the soil increased significantly compared with CK, and the change trend was similar to that of OBC. With the increase of incubation time, the catalase activity first increased to reach the highest value at 30th day and then decreased and then stabilized. At the end of incubation, 1% OBC-Mg, 2% OBC-Mg, and 4% OBC-Mg increased by 73.33, 100.00, and 116.67%, respectively, and the catalase activity increased with the increase of applied percentage.

#### 3.5.2. Impact of OBC and OBC-Mg on soil urease activity

**Figure 13:**
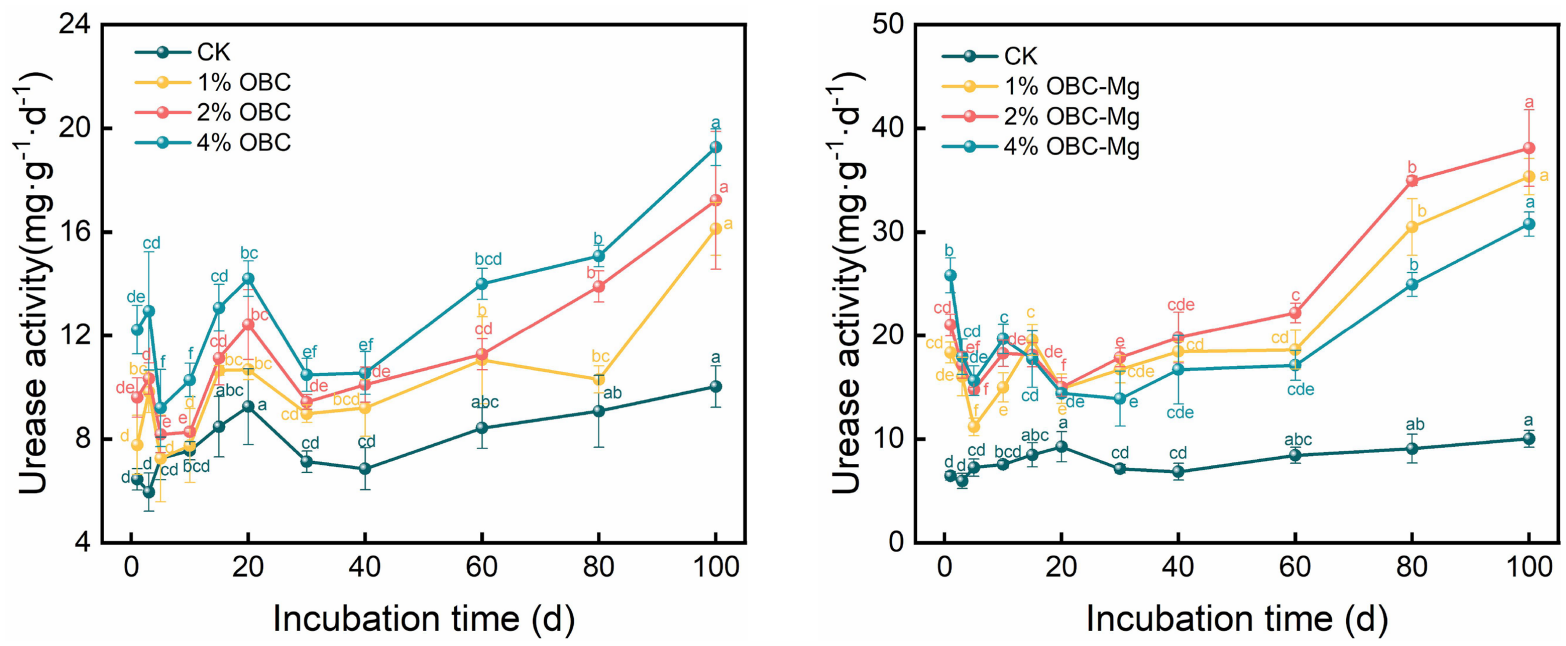
Changes in soil urease activity after the amendment of OBC and OBC-Mg.

As shown in Figure 13, the application of OBC and OBC-Mg to citrus orchard soils significantly increased the urease activity in the soil. After the application of OBC, the overall trend of increasing urease activity of each proportion with incubation time was observed, and at the end of incubation, the urease activity of each proportion was higher than that of CK, 1% OBC was 1.61 times, 2% OBC was 1.72 times, and 4% OBC was 1.92 times than that of CK. Throughout the incubation period, the urease activity of each applied ratio showed 4% OBC > 2% OBC > 1% OBC > CK. After the application of OBC-Mg, the urease activity of each ratio gradually increased after decreasing from 0 to 5 days. At the end of the incubation, the urease activity increased by 252.49% for 1% OBC-Mg, 279.91% for 2% OBC-Mg and 206.80% for 4% OBC-Mg compared to CK. The urease activity of each treatment showed 4% OBC-Mg > 2% OBC-Mg > 1% OBC-Mg > CK on 0–10 days of incubation, and the urease activity of each applied percentage showed 2% OBC-Mg > 1% OBC-Mg > 4% OBC > CK after 20 days of incubation.

#### 3.5.3. Impact of OBC and OBC-Mg on soil sucrase activity

**Figure 14:**
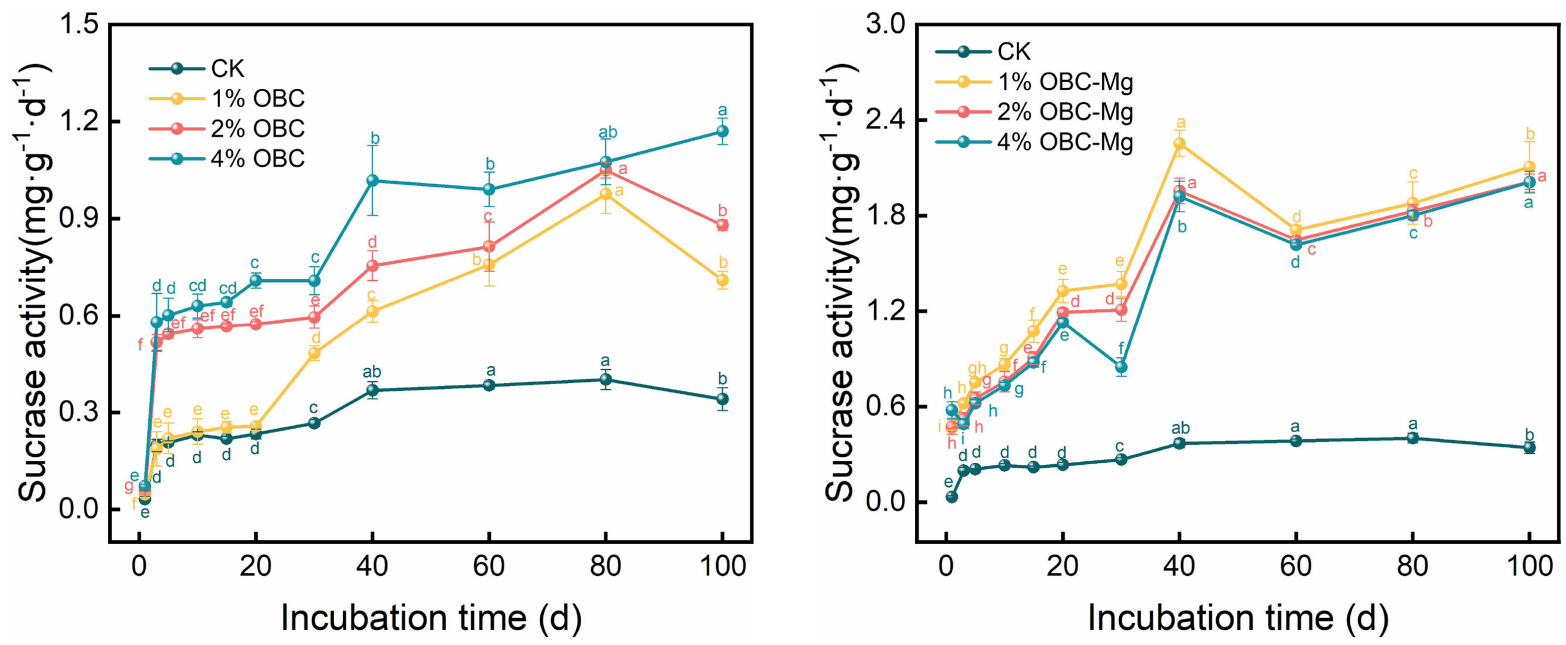
Changes in soil sucrase activity after the amendment of OBC and OBC-Mg.

As shown in Figure 14, the application of OBC and OBC-Mg to citrus orchard soils increased the sucrase activity in the soil. The application of OBC showed an overall trend of increasing sucrase activity with incubation time. At the end of incubation, the urease activity was higher than that of CK for all proportions, with an increase of 108.21 for 1% OBC, 158.03 for 2% OBC, and 243.05% for 4% OBC compared to CK. The sucrase activity of each treatment increased with the applied percentage throughout the incubation period. After the application of OBC-Mg, the sucrase activity increased with the increase of incubation time for each percentage. At the end of incubation, such enzyme activity increased by 517.60% for 1% OBC-Mg, 490.18% for 2% OBC-Mg and 489.65% for 4% OBC-Mg compared to CK. The sucrase activity of each ratio decreased with the increase of applied ratio, 1% OBC-Mg > 2% OBC-Mg > 4% OBC > CK.

### 3.6. Correlation analysis

**Figure 15:**
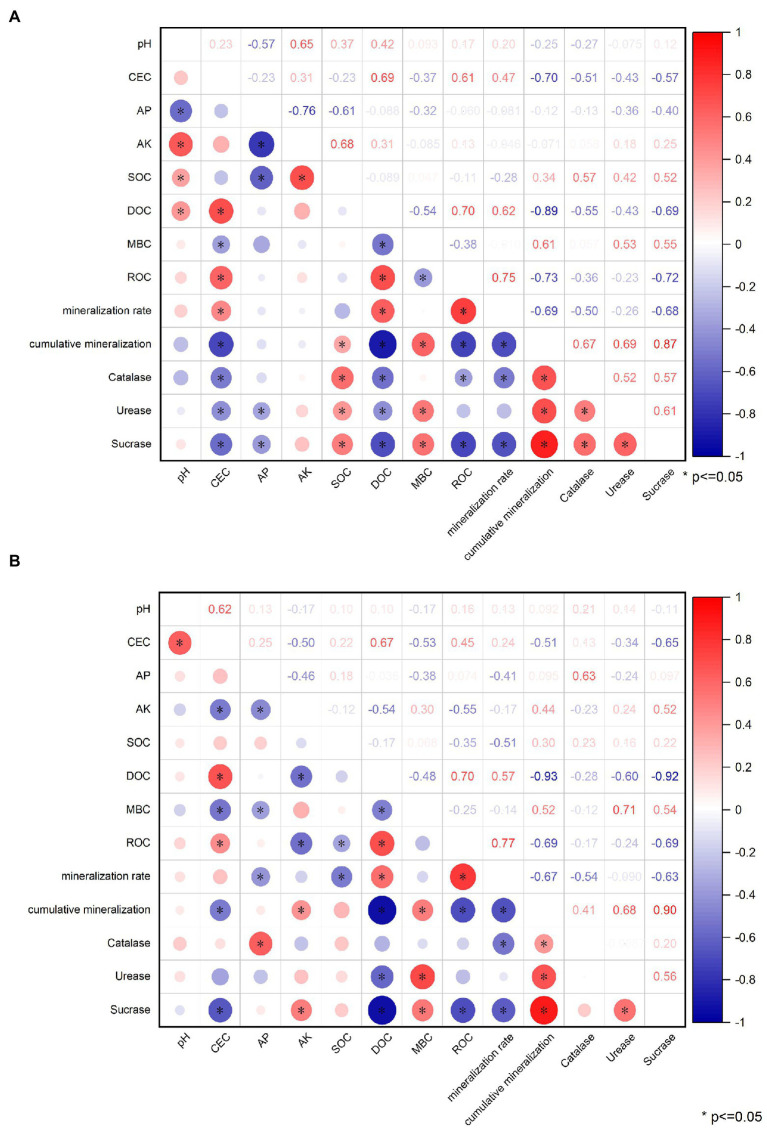
Correlation between physical and chemical properties, carbon composition, enzyme activity and mineralization of **(A)** OBC-Mg and **(B)** OBC-Mg.

In Figure 15A, it can be seen that after applying OBC to citrus orchard soils, soil pH was significantly positively correlated (*p* < = 0.05) with AK, SOC and DOC, and negatively correlated with AP. Soil CEC content was positively correlated with DOC, ROC, and organic carbon mineralization rate. It was negatively correlated with MBC, cumulative mineralization of organic carbon, catalase, urease, and sucrase. AP content was negatively correlated with AK, SOC, urease and sucrase. DOC was positively correlated with ROC and organic carbon mineralization rate, and negatively correlated with MBC, organic carbon mineralization, catalase, urease and sucrase. MBC was positively correlated with organic carbon mineralization, urease and sucrase, and negatively correlated with ROC. The ROC was positively correlated with the rate of organic carbon mineralization and negatively correlated with the cumulative mineralization of organic carbon, catalase and sucrase. The rate of organic carbon mineralization was negatively correlated with the cumulative mineralization of organic carbon, catalase, and sucrase. Cumulative mineralization of organic carbon was positively correlated with catalase, urease and sucrase. Catalase was positively correlated with urease and sucrase. Urease was positively correlated with sucrase.

As seen in Figure 15B, soil pH was significantly and positively correlated (*p* < = 0.05) with CEC after application of OBC-Mg to citrus orchard soil. Soil CEC content was positively correlated with DOC and ROC, and negatively correlated with AK, MBC, accumulated organic carbon mineralization and sucrose. AP was positively correlated with catalase and negatively correlated with AK, MBC and organic carbon mineralization rate. SOC was negatively correlated with ROC and organic carbon mineralization rate. DOC was positively correlated with ROC and organic carbon mineralization rate, and negatively correlated with MBC, organic carbon mineralization, urease and sucrase. The ROC was positively correlated with the rate of organic carbon mineralization and negatively correlated with the cumulative mineralization of organic carbon and sucrase. The organic carbon mineralization rate was negatively correlated with the accumulated organic carbon mineralization, catalase, and sucrase. The cumulative mineralization of organic carbon was positively correlated with catalase, urease and sucrase. Meanwhile, urease was positively correlated with sucrase.

## 4. Discussion

### 4.1. Effect of biochar on soil physicochemical properties

The application of OBC and OBC-Mg significantly increased soil pH compared to CK. The increase in pH after OBC application may be due to the fact that biochar contains soluble organic and inorganic bases, making it highly alkaline (Fidel et al., 2017; Shi et al., 2019). Applying biochar to acidic soil neutralized soil acidity, thus increasing soil pH. Whereas the increase in pH after OBC-Mg application was greater than that of OBC, probably due to the fact that the Mg-modified biochar surface was loaded with more Mg^2+^ and other alkaline cations, which are converted to oxides, hydroxides and carbonates after pyrolysis, resulting in higher pH of Mg-modified biochar and thus better increase in soil pH. Similarly, Khan et al. (2022) used pristine and Mg-modified rice straw biochar applied to the soil and an increase in soil pH occurred, which is consistent with the results of this study. In this study, the application of OBC and OBC-Mg to citrus soils significantly increased soil pH, which was mainly due to the alkalinity of biochar, indicating that both applications could improve soil acidity.

Cation exchange capacity (CEC) reflects the amount of negative charge in the soil that can be neutralized by exchangeable cations such as Mg and Ca (Wu et al., 2020). Application of OBC-Mg to citrus orchard soils increased the CEC content of the soil, partly because of the adsorption of oxides such as Al and Fe by biochar and the decrease in the zero point charge of the soil (Hailegnaw et al., 2019), and partly because of the presence of oxygen-containing functional groups on the surface of biochar (Yadav et al., 2019). The application of OBC to citrus orchard soils reduced the CEC content of the soil, and a similar phenomenon was found in the study of Seokjoon’s (2005), which suggested that the reduction of CEC after biochar application might be mainly due to the enrichment of humic and xanthic acids in the soil by organic matter, thus blocking the internal pores of the biochar from the next physical sorption step and thus reducing the CEC. OBC and OBC-Mg applied to the soil differed in their effect on cation exchange mainly due to the higher Mg content in OBC-Mg, which can neutralize more with negative charges and increase soil CEC. The effect of OBC-Mg on pH and CEC in this study was significantly positively correlated, with higher pH and higher CEC in OBC-Mg soil, which is consistent with the findings of Heikkinen et al. (2019). Also correlation analysis showed that soil CEC was significantly negatively correlated with cumulative mineralization, indicating that the elevated cumulative mineralization of soil organic carbon by OBC and OBC-Mg applied to the soil led to a decrease in CEC.

A study by Li, S. et al. (2018) found that the application of biochar to the soil was effective in reducing N leaching and increasing K content. The application of OBC and OBC-Mg resulted in a significant increase in AP content, which is consistent with the findings of other studies. Kamran et al. (2018) applied three types of biochar, chicken manure bochar, pig manure biochar and peat moss biochar to soil, the results showed a significant increase in soil AP, and the change in AP content was related to the amount of biochar applied. The increase in AP was due to three reasons. On the one hand, biochar contains a certain amount of phosphorus itself and thus directly increases soil effective phosphorus (Hong and Lu, 2018). On the other hand, biochar increases soil pH and changes the activity of cations such as Al^3+^, Fe^3+^, and Ca^2+^, thus reducing P adsorption or increasing P desorption, making phosphorus more effective (Yang and Lu, 2022). Finally, because biochar applied to the soil, causing changes in the soil microbial environment and increasing the phosphorus fixation capacity of the soil (Tian et al., 2021). The main reason of the decrease in AP content with increasing incubation time is that the fixation of phosphorus by the soil leads to a decrease in effective phosphorus (Kahura et al., 2018). The application of alkaline biochar in acidic soils can better improve the bioeffectiveness of phosphorus (Chintala et al., 2014), and the difference in the effect of OBC and OBC-Mg application to soil on AP content may be due to the inconsistent chemical composition and surface characteristics of biochar. In this study, the application of OBC-Mg could better increase the content of fast-acting phosphorus, mainly because OBC-Mg was loaded with more cations on the surface, which increased the effectiveness of phosphorus. Furthermore, it is also reported that biochar can improve the relative abundance of proteobacteria in soil which plays a significant role in improving soil properties (Zhang et al., 2022). Correlation analysis showed that AP content was significantly and negatively correlated with AK content, indicating that AP content increased with the decrease of AK in the soil.

Citrus peel biochar (OBC) application to soil significantly increased soil AK content and increased with increasing application rate. This is consistent with the findings of Yao et al. (2021), which found a significant increase in AK after biochar application in heavily salinized paddy fields. The increase in AK content may be due to the interaction of biochar with soil minerals that affects the release of nutrients from the soil, which results in an increase in AK (El-Naggar et al., 2019). Analysis of the EDS of biochar showed that the K content of Mg-modified biochar was significantly lower, which led to a better increase of the AK content by OBC, while the effect of OBC-Mg on AK was smaller.

### 4.2. Effect of biochar on soil organic carbon mineralization in citrus orchards

The stimulatory effect of biochar on soil CO_2_ emissions is governed by various factors, such as biochar physicochemical properties, biochar stability and soil properties (Song et al., 2019), and microorganisms in biochar may also affect soil CO_2_ emissions (Wang, L. et al., 2021). Some studies have shown that, the more biochar applied, the faster respiration rate of soil microorganisms was and the more total CO_2_ released at the beginning of incubation (Steiner et al., 2008). And when the amount of biochar applied is higher, soil microorganisms are more likely to decompose water-soluble organic matter in biochar for microbial activity, thus releasing more CO_2_ (Zhang et al., 2020). The rate of soil organic carbon mineralization in different treatments has a similar pattern. And in this study, the application of OBC and OBC-Mg in citrus orchard soil promoted CO_2_ emission in the early incubation period, then decreased and stabilized with increasing incubation time, it is consistent with the findings of Ameloot et al. (2013). This is due to the application of biochar to the soil, which stimulates the native carbon pool, and the stimulation of soil organisms leading to the biodegradation of biochar components, resulting in enhanced CO_2_ release (Blagodatskaya and Kuzyakov, 2008). Similarly, Orlova et al. (2019) made biochar from birch and poplar wood at 550°C and injected it into the soil, showing that 1% biochar increased mineralization by 15–18%, while a study by El-Naggar et al. (2018) showed that rice straw biochar and sludge biochar promoted mineralization for 3 and 1.5 months. Whereas CO_2_ emissions decreased in the later stages of incubation due to biochar, which added higher carbon content and richer aromatic structure, thus enhancing resistance to biodegradation and ultimately leading to a potential negative excitation effect. Also, the inhibition of soil mineralization by biochar generally occurred in the later stages of incubation (Rasul et al., 2022), which is consistent with our findings. Similar effective reduction of soil CO_2_ emissions by biochar has been reported in other studies (Hua et al., 2014; Rubin et al., 2020). The cumulative mineralization of organic carbon was significantly and positively correlated with catalase, urease, and sucrase activities. In this study, the stimulation of microbial activity in the soil led to the increase in soil organic carbon mineralization, which resulted in an increase in soil enzyme activity.

### 4.3. Effect of biochar on different carbon fractions in citrus orchard soils

The application of OBC and OBC-Mg to citrus orchard soils not only increased soil SOC content, but also showed a positive correlation with the percentage of biochar application, which is consistent with the findings of Novak et al. (2010). The increase in soil SOC content may be due to the high carbon content of biochar itself, and the application of biochar to soil is equivalent to the application of exogenous organic carbon (Li, Y. et al., 2018). The refractory nature of biochar allows it to persist in the soil, which contributes to the increase in organic carbon (Rasul et al., 2022). Similarly, Dong et al. (2018) applied different amounts of rice husk and cotton seed hull biochar to the soil to observe organic carbon changes and showed that biochar significantly increased SOC content and SOC increased with increasing biochar amount.

Soil microbial biomass carbon (MBC), DOC, and ROC are reactive organic carbon in soils and usually respond rapidly to soil changes (Wang, X. et al., 2021). Soil MBC is derived from the hydrolysis of soil organic matter, soil microorganisms themselves and their metabolites (Zhang et al., 2018). In this study, application of OBC and OBC-Mg to citrus orchard soil increased or decreased MBC content compared to CK. The decrease in MBC in the early stages of incubation was mainly due to the depletion and mineralization of unstable components of organic carbon, resulting in a decrease in MBC (Huang et al., 2021). While the gradual increase in MBC in the later stages may be due to a stable and less disturbed soil environment in the later stages, where microbial activity increases leading to an increase in MBC. In this study, the MBC was significantly and positively correlated with the cumulative mineralization of organic carbon, indicating that the mineralization of soil organic carbon also affects the MBC.

Soil dissolved organic carbon (DOC) is the most active component of SOC and an important source of organic carbon and an important indicator of soil microbial effectiveness (Hu et al., 2022). In this study, application of OBC and OBC-Mg to citrus orchard soil increased DOC content and showed an overall decreasing trend with increasing incubation time. The increase in DOC content may be due to the release of the active organic carbon fraction from biochar into the soil after biochar application. This induced the conversion of SOC to DOC, thus leading to an increase in soil DOC content (Ye and Horwath, 2017). And the decrease in DOC content with increasing incubation time is because the stabilized organic carbon from biochar makes the soil SOC increase. And the decomposed active organic carbon, leading to a decrease in DOC content (Zhang et al., 2017). Compared with OBC, OBC-Mg has a better effect on the enhancement of soil DOC content mainly because OBC-Mg has a better effect on the improvement of soil acidity. And the increase of soil pH leads to the deprotonation process of weakly acidic functional groups in soluble organic carbon molecules. This increases the surface charge density of soil soluble organic carbon molecules and enhances hydrophilicity, promoting the solubilization of soil soluble organic carbon (Smebye et al., 2016). In this study, DOC content was significantly and negatively correlated with the accumulated mineralization of organic carbon.

Soil readily oxidized organic carbon (ROC) is the more reactive and easily oxidized carbon fraction of the organic carbon pool (Yang et al., 2017). In this study, OBC and OBC-Mg increased the ROC content in the soil compared to CK, and the ROC content decreased with increasing incubation time. ROC is the same active organic carbon as DOC, and the reason for its change is similar to DOC. Biochar applied to citrus orchard soil increased the soil microbial activity leading to an increase in ROC (Abiven et al., 2015). With increasing incubation time, the active organic carbon of biochar was decomposed and the ROC content decreased. In this study, the ROC content was significantly and negatively correlated with the cumulative mineralization of organic carbon. The ROC content decreased as the cumulative mineralization of organic carbon increased, which is consistent with the changes in DOC in the previous paper. This indicated that the active organic carbon components such as soil DOC and ROC are susceptible to the cumulative mineralization of organic carbon.

### 4.4. Effect of biochar on soil enzyme activity in citrus orchards

Soil enzyme activity is an important biological indicator of soil quality, and changes in microbial activity are mainly due to changes in the physicochemical properties of the soil. Biochar surface has a high potential to adsorb organic molecules, including enzymes and substrates, thus altering enzyme activity (Foster et al., 2018). Application of OBC and OBC-Mg to citrus orchard soil resulted in a significant increase in soil enzyme activity compared to the CK. Catalase is an important enzyme that indicates the redox potential of soil (Yang et al., 2016). And biochar as an additional carbon source effectively improves the survival environment of microorganisms in soil by providing more nutrients and increasing the number and activity of microorganisms, which leads to more secretion of catalase by microorganisms. Masto et al. (2013b) applied water hyacinth biochar to soil, the results showed that catalase activity increased with the amount of biochar applied, which is consistent with the findings of this study. The effect of OBC on soil catalase was less than that of OBC-Mg because catalase is stable and not easily affected by environmental conditions, but OBC-Mg caused changes in the physicochemical properties of biochar and increased the impact on the soil environment (Yang et al., 2016). The catalase activity of the 1% OBC and 2% OBC fractions was lower than that of CK on 0–30 days of incubation, which may be due to the large availability of soil organic matter and microbial community in the pre-culture period, resulting in lower catalase activity (Ullah et al., 2020). Soil urease catalyzes the hydrolysis of phthalate bonds in organic molecules and promotes the conversion of soil organic N to active N (Gao et al., 2017). Application of OBC and OBC-Mg significantly increased urease activity compared to CK because urease activity is associated with microbial growth and the application of biochar stimulated soil microbial activity, thus leading to an increase in soil urease activity (Yadav et al., 2019). Jing et al. (2020) studied the application of three types of straw biochar from wheat, rice, and maize to soil and showed an increase in urease activity of 42.5–94.2%. Both biochar applied to soil also increased soil sucrase activity, this is because sucrose can be broken down by sucrase into glucose and fructose. Glucose and fructose are important carbon source for microorganisms, and the higher carbon to nitrogen ratio in soil is, the more enzyme substrate it can provide, thus increasing sucrase activity (Tang et al., 2022). Meanwhile, catalase, urease and sucrase activities were all significantly and positively correlated with the cumulative mineralization of soil organic carbon, and soil enzyme activity increased with the increase in cumulative mineralization of organic carbon.

## 5. Conclusion

In this study, we investigated the effects of OBC and OBC-Mg on soil organic carbon mineralization in citrus orchards by applying them to citrus orchard soils at different rate. The results showed that there was a significant difference between OBC and OBC-Mg on soil organic carbon mineralization, and OBC-Mg significantly increased soil organic carbon content with a significantly higher enhancement effect than OBC. 1% OBC-Mg reduced soil organic carbon mineralization and had good potential for soil carbon sequestration and reduction. Meanwhile, with the increase of cumulative soil organic carbon mineralization, OBC-Mg treatment more significantly increased the activities of soil catalase, urease and sucrase than OBC treatment. In conclusion, Mg-modified citrus peel biochar had better carbon sequestration effect on citrus orchard soil. We will continue our metagenomic measurements and conduct an in-depth mechanistic study on the application of Mg-modified citrus peel biochar to citrus soil.

## Data availability statement

The raw data supporting the conclusions of this article will be made available by the authors, without undue reservation.

## Author contributions

LH: conceptualization and data curation. RH: software, writing original draft preparation and data curation. LZ, RQ, and XH: data curation and supervision. HD: writing review and editing. KL: funding acquisition. All authors contributed to the article and approved the submitted version.

## Funding

This work was supported by Guangxi Surface project, grant no. 2022GXNSFAA035555. The Key Laboratory of Ecology of Rare and Endangered Species and Environmental Protection (Guangxi Normal University), Ministry of Education, China, grant no. ERESEP2022Z13; Open Fund of Key Laboratory of Geospatial Technology for the Middle and Lower Yellow River Regions (Henan University), Ministry of Education, grant no. GTYR202103; Guangxi Key Research and development Program, grant no. AB22080097.

## Conflict of interest

The authors declare that the research was conducted in the absence of any commercial or financial relationships that could be construed as a potential conflict of interest.

## Publisher’s note

All claims expressed in this article are solely those of the authors and do not necessarily represent those of their affiliated organizations, or those of the publisher, the editors and the reviewers. Any product that may be evaluated in this article, or claim that may be made by its manufacturer, is not guaranteed or endorsed by the publisher.

## References

[ref1] Abhishek K. Shrivastava A. Vimal V. Gupta A. K. Bhujbal S. K. Biswas J. K. (2022). Biochar application for greenhouse gas mitigation, contaminants immobilization and soil fertility enhancement: a state-of-the-art review. Sci. Total Environ. 853:158562. doi: 10.1016/j.scitotenv.2022.158562, PMID: 36089037

[ref2] Abiven S. Hund A. Martinsen V. Cornelissen G. (2015). Biochar amendment increases maize root surface areas and branching: a shovelomics study in Zambia. Plant Soil 395, 45–55. doi: 10.1007/s11104-015-2533-2

[ref3] Ameloot N. Graber E. R. Verheijen F. G. A. De Neve S. (2013). Interactions between biochar stability and soil organisms: review and research needs. Eur. J. Soil Sci. 64, 379–390. doi: 10.1111/ejss.12064

[ref4] Bashir S. Hussain Q. Zhu J. Fu Q. Houben D. Hu H. (2020). Efficiency of KOH-modified rice straw-derived biochar for reducing cadmium mobility, bioaccessibility and bioavailability risk index in red soil. Pedosphere 30, 874–882. doi: 10.1016/S1002-0160(20)60043-1

[ref5] Blagodatskaya Е. Kuzyakov Y. (2008). Mechanisms of real and apparent priming effects and their dependence on soil microbial biomass and community structure: critical review. Biol. Fertil. Soils 45, 115–131. doi: 10.1007/s00374-008-0334-y

[ref6] Chen W. Meng J. Han X. Lan Y. Zhang W. (2019). Past, present, and future of biochar. Biochar. 1, 75–87. doi: 10.1007/s42773-019-00008-3

[ref7] Chen H. Wang Y. Zhang L. Luo L. Ye X. Li Y. (2019). Advances in magnesium nutritional status and its mechanisms of physiological and molecule in citrus. J. Fruit Sci. 36, 1578–1590. doi: 10.13925/j.cnki.gsxb.20190210

[ref8] Chintala R. Schumacher T. E. McDonald L. M. Clay D. E. Malo D. D. Papiernik S.-K. (2014). Phosphorus sorption and availability from biochars and soil/biochar mixtures. CLEAN Soil Air Water 42, 626–634. doi: 10.1002/clen.201300089, PMID: 29426221

[ref9] Deng H. Zhang J. Huang R. Wang W. Meng M. Hu L. (2022). Adsorption of malachite green and Pb^2+^ by KMnO_4_^−^Modified biochar: insights and mechanisms. Sustainability 14. doi: 10.3390/su14042040

[ref10] Dong X. Singh B. P. Li G. Lin Q. Zhao X. (2018). Biochar application constrained native soil organic carbon accumulation from wheat residue inputs in a long-term wheat-maize cropping system. Agric. Ecosyst. Environ. 252, 200–207. doi: 10.1016/j.agee.2017.08.026

[ref11] Du Y. Guo X. Li J. Liu Y. Luo J. Liang Y. (2022). Elevated carbon dioxide stimulates nitrous oxide emission in agricultural soils: a global meta-analysis. Pedosphere 32, 3–14. doi: 10.1016/S1002-0160(21)60057-7, PMID: 34915007

[ref12] El-Naggar A. El-Naggar A. H. Shaheen S. M. Sarkar B. Chang S. X. Tsang D. C. W. (2019). Biochar composition-dependent impacts on soil nutrient release, carbon mineralization, and potential environmental risk: a review. J. Environ. Manag. 241, 458–467. doi: 10.1016/j.jenvman.2019.02.044, PMID: 31027831

[ref13] El-Naggar A. Lee S. S. Awad Y. M. Yang X. Ryu C. Rizwan M. (2018). Influence of soil properties and feedstocks on biochar potential for carbon mineralization and improvement of infertile soils. Geoderma 332, 100–108. doi: 10.1016/j.geoderma.2018.06.017

[ref14] Fidel R. B. Laird D. A. Thompson M. L. Lawrinenko M. (2017). Characterization and quantification of biochar alkalinity. Chemosphere 167, 367–373. doi: 10.1016/j.chemosphere.2016.09.151, PMID: 27743533

[ref15] Foster E. Fogle E. Cotrufo M. (2018). Sorption to biochar impacts β-glucosidase and phosphatase enzyme activities. Agriculture 8. doi: 10.3390/agriculture8100158, PMID: 32438238

[ref16] Gan H. Y. Schöning I. Schall P. Ammer C. Schrumpf M. (2020). Soil organic matter mineralization as driven by nutrient stoichiometry in soils under differently managed Forest stands. Front. For. Global Change 3:99. doi: 10.3389/ffgc.2020.00099

[ref17] Gao S. Hoffman-Krull K. DeLuca T. H. (2017). Soil biochemical properties and crop productivity following application of locally produced biochar at organic farms on Waldron Island. Biogeochemistry 136, 31–46. doi: 10.1007/s10533-017-0379-9

[ref18] Hailegnaw N. S. Mercl F. Pračke K. Száková J. Tlustoš P. (2019). Mutual relationships of biochar and soil pH, CEC, and exchangeable base cations in a model laboratory experiment. J. Soils Sediments 19, 2405–2416. doi: 10.1007/s11368-019-02264-z

[ref19] Heikkinen J. Keskinen R. Soinne H. Hyväluoma J. Nikama J. Wikberg H. (2019). Possibilities to improve soil aggregate stability using biochars derived from various biomasses through slow pyrolysis, hydrothermal carbonization, or torrefaction. Geoderma 344, 40–49. doi: 10.1016/j.geoderma.2019.02.028, PMID: 36610482

[ref20] Hong C. Lu S. (2018). Does biochar affect the availability and chemical fractionation of phosphate in soils? Environ. Sci. Pollut. Res. Int. 25, 8725–8734. doi: 10.1007/s11356-018-1219-8, PMID: 29327187

[ref21] Hu L. Huang R. Deng H. Li K. Peng J. Zhou L. (2022). Effects of different intercropping methods on soil organic carbon and aggregate stability in sugarcane field. Pol. J. Environ. Stud. 31, 3587–3596. doi: 10.15244/pjoes/147187

[ref22] Hu L. Li S. Li K. Huang H. Wan W. Huang Q. (2020). Effects of two types of straw biochar on the mineralization of soil organic carbon in farmland. Sustainability 12. doi: 10.3390/su122410586

[ref23] Hua L. Lu Z. Ma H. Jin S. (2014). Effect of biochar on carbon dioxide release, organic carbon accumulation, and aggregation of soil. Environ. Prog. Sustain. Energy 33, 941–946. doi: 10.1002/ep.11867

[ref24] Huang R. Lan T. Song X. Li J. Ling J. Deng O. (2021). Soil labile organic carbon impacts C:N:P stoichiometry in urban park green spaces depending on vegetation types and time after planting. Appl. Soil Ecol. 163:103926. doi: 10.1016/j.apsoil.2021.103926

[ref25] Huang R. Tian D. Liu J. Lv S. He X. Gao M. (2018). Responses of soil carbon pool and soil aggregates associated organic carbon to straw and straw-derived biochar addition in a dryland cropping mesocosm system. Agric. Ecosyst. Environ. 265, 576–586. doi: 10.1016/j.agee.2018.07.013

[ref26] Jiao Y. Wang T. He M. Liu X. Lin C. Ouyang W. (2022). Simultaneous stabilization of Sb and as co-contaminated soil by FeMg modified biochar. Sci. Total Environ. 830:154831. doi: 10.1016/j.scitotenv.2022.154831 35346707

[ref27] Jien S. H. Chen W. C. Ok Y. S. Awad Y. M. Liao C. S. (2018). Short-term biochar application induced variations in C and N mineralization in a compost-amended tropical soil. Environ. Sci. Pollut. Res. Int. 25, 25715–25725. doi: 10.1007/s11356-017-9234-8, PMID: 28573558

[ref28] Jing Y. Zhang Y. Han I. Wang P. Mei Q. Huang Y. (2020). Effects of different straw biochars on soil organic carbon, nitrogen, available phosphorus, and enzyme activity in paddy soil. Sci. Rep. 10:8837. doi: 10.1038/s41598-020-65796-2 32483277 PMC7264176

[ref29] Kahura M. W. Min H. Kim M. S. Kim J. G. (2018). Assessing phosphorus availability in a high pH, biochar amended soil under inorganic and organic fertilization. Ecol. Resilient Infrastruct. 5, 11–18. doi: 10.1016/j.scitotenv.2022.158562

[ref30] Kamran M. A. Jiang J. Li J. Y. Shi R. Y. Mehmood K. Baquy M. A. A. (2018). Amelioration of soil acidity, Olsen-P, and phosphatase activity by manure- and peat-derived biochars in different acidic soils. Arab. J. Geosci. 11:272. doi: 10.1007/s12517-018-3616-1

[ref31] Khan M. N. Li D. Shah A. Huang J. Zhang L. Nunez-Delgado A. (2022). The impact of pristine and modified rice straw biochar on the emission of greenhouse gases from a red acidic soil. Environ. Res. 208:112676. doi: 10.1016/j.envres.2022.112676, PMID: 34998810

[ref32] Korai P. K. Xia X. Liu X. Bian R. Omondi M. O. Nahayo A. (2018). Extractable pool of biochar controls on crop productivity rather than greenhouse gas emission from a rice paddy under rice-wheat rotation. Sci. Rep. 8:802. doi: 10.1038/s41598-018-19331-z 29339780 PMC5770379

[ref33] Kuo Y. L. Lee C. H. Jien S. H. (2020). Reduction of nutrient leaching potential in coarse-textured soil by using biochar. Water 12:2012. doi: 10.3390/w12072012

[ref34] Li Y. Li Y. Chang S. X. Yang Y. Fu S. Jiang P. (2018). Biochar reduces soil heterotrophic respiration in a subtropical plantation through increasing soil organic carbon recalcitrancy and decreasing carbon-degrading microbial activity. Soil Biol. Biochem. 122, 173–185. doi: 10.1016/j.soilbio.2018.04.019

[ref35] Li R. Wang J. J. Zhou B. Awasthi M. K. Ali A. Zhang Z. (2016). Enhancing phosphate adsorption by mg/Al layered double hydroxide functionalized biochar with different mg/Al ratios. Sci. Total Environ. 559, 121–129. doi: 10.1016/j.scitotenv.2016.03.151 27058131

[ref36] Li R. Wang J. J. Zhou B. Zhang Z. Liu S. Lei S. (2017). Simultaneous capture removal of phosphate, ammonium and organic substances by MgO impregnated biochar and its potential use in swine wastewater treatment. J. Clean. Prod. 147, 96–107. doi: 10.1016/j.jclepro.2017.01.069

[ref37] Li S. Zhang Y. Yan W. Shangguan Z. (2018). Effect of biochar application method on nitrogen leaching and hydraulic conductivity in a silty clay soil. Soil Tillage Res. 183, 100–108. doi: 10.1016/j.still.2018.06.006

[ref38] Liu Z. Tang J. Ren X. Schaeffer S. M. (2021). Effects of phosphorus modified nZVI-biochar composite on emission of greenhouse gases and changes of microbial community in soil. Environ. Pollut. 274:116483. doi: 10.1016/j.envpol.2021.116483, PMID: 33508717

[ref39] Lu Y. Silveira M. L. O'Connor G. A. Vendramini J. M. B. Erickson J. E. Li Y. C. (2020). Biochar impacts on nutrient dynamics in a subtropical grassland soil: 1. Nitrogen and phosphorus leaching. J. Environ. Qual. 49, 1408–1420. doi: 10.1002/jeq2.20139, PMID: 33016442

[ref40] Masto R. E. Ansari M. A. George J. Selvi V. A. Ram L. C. (2013a). Co-application of biochar and lignite fly ash on soil nutrients and biological parameters at different crop growth stages of Zea mays. Ecol. Eng. 58, 314–322. doi: 10.1016/j.ecoleng.2013.07.011

[ref41] Masto R. E. Kumar S. Rout T. K. Sarkar P. George J. Ram L. C. (2013b). Biochar from water hyacinth (Eichornia crassipes) and its impact on soil biological activity. Catena 111, 64–71. doi: 10.1016/j.catena.2013.06.025

[ref42] Novak J. M. Busscher W. J. Watts D. W. Laird D. A. Ahmedna M. A. Niandou M. A. S. (2010). Short-term CO_2_ mineralization after additions of biochar and switchgrass to a typic Kandiudult. Geoderma 154, 281–288. doi: 10.1016/j.geoderma.2009.10.014

[ref43] Oo A. Z. Sudo S. Akiyama H. Win K. T. Shibata A. Yamamoto A. (2018). Effect of dolomite and biochar addition on N_2_O and CO_2_ emissions from acidic tea field soil. PLoS One 13:e0192235. doi: 10.1371/journal.pone.0192235, PMID: 29394272 PMC5796709

[ref44] Orlova N. Abakumov E. Orlova E. Yakkonen K. Shahnazarova V. (2019). Soil organic matter alteration under biochar amendment: study in the incubation experiment on the podzol soils of the Leningrad region (Russia). J. Soils Sediments 19, 2708–2716. doi: 10.1007/s11368-019-02256-z

[ref45] Pei J. Zhuang S. Cui J. Li J. Li B. Wu J. (2017). Biochar decreased the temperature sensitivity of soil carbon decomposition in a paddy field. Agric. Ecosyst. Environ. 249, 156–164. doi: 10.1016/j.agee.2017.08.029

[ref46] Perez-Quezada J. F. Urrutia P. Olivares-Rojas J. Meijide A. Sánchez-Cañete E. P. (2021). Long term effects of fire on the soil greenhouse gas balance of an old-growth temperate rainforest. Sci. Total Environ. 755:142442. doi: 10.1016/j.scitotenv.2020.142442, PMID: 33022457

[ref47] Qiao J. Li X. Shi R. (2021). Research on the industry of three citrus producing areas in China – taking Guangxi, Hunan and Hubei as examples. Yunnan Sci. Technol. Manag. 01, 45–49. doi: 10.19774/j.cnki.53-1085.2021.01.012

[ref48] Rasul M. Cho J. Shin H. S. Hur J. (2022). Biochar-induced priming effects in soil via modifying the status of soil organic matter and microflora: a review. Sci. Total Environ. 805:150304. doi: 10.1016/j.scitotenv.2021.150304 34536873

[ref49] Rong G. Zhang X. Wu H. Ge N. Yao Y. Wei X. (2021). Changes in soil organic carbon and nitrogen mineralization and their temperature sensitivity in response to afforestation across China’s loess plateau. Catena 202:105226. doi: 10.1016/j.catena.2021.105226

[ref50] Rubin R. L. Anderson T. R. Ballantine K. A. (2020). Biochar simultaneously reduces nutrient leaching and greenhouse gas emissions in restored wetland soils. Wetlands 40, 1981–1991. doi: 10.1007/s13157-020-01380-8

[ref51] Sarma B. Borkotoki B. Narzari R. Kataki R. Gogoi N. (2017). Organic amendments: effect on carbon mineralization and crop productivity in acidic soil. J. Clean. Prod. 152, 157–166. doi: 10.1016/j.jclepro.2017.03.124, PMID: 36129010

[ref52] Seokjoon K. (2005). Effect of natural organic substances on the surface and adsorptive properties of environmental black carbon (char): pseudo pore blockage by model lipid components and its implications for N2-probed surface properties of natural sorbents. Environ. Sci. Technol. 39, 7932–7939. doi: 10.1021/es050976h, PMID: 16295858

[ref53] Shan R. Li W. Chen Y. Sun X. (2022). Effects of mg-modified biochar on the bioavailability of cadmium in soil. Bioresources 15, 8008–8025. doi: 10.15376/biores.15.4.8008-8025

[ref54] Shi R. Y. Li J. Y. Ni N. Xu R. K. (2019). Understanding the biochar's role in ameliorating soil acidity. J. Integr. Agric. 18, 1508–1517. doi: 10.1016/S2095-3119(18)62148-3, PMID: 34202337

[ref55] Smebye A. Alling V. Vogt R. D. Gadmar T. C. Mulder J. (2016). Cornelissen G. Biochar amendment to soil changes dissolved organic matter content and composition. Chemosphere 142, 100–105, doi: 10.1016/j.chemosphere.2015.04.087, PMID: . 25980657

[ref1001] Song Y. Li Y. Cai Y. Fu S. Luo Y. Wang H. (2019). Biochar decreases soil N_2_O emissions in Moso bamboo plantations through decreasing labile N concentrations, N-cycling enzyme activities and nitrification/denitrification rates. Geoderma 348, 135–145. doi: 10.1016/j.geoderma.2019.04.025

[ref56] Stefaner K. Ghosh S. Mohd Yusof M. L. Ibrahim H. Leitgeb E. Schindlbacher A. (2021). Soil greenhouse gas fluxes from a humid tropical forest and differently managed urban parkland in Singapore. Sci. Total Environ. 786:147305. doi: 10.1016/j.scitotenv.2021.147305

[ref57] Steiner C. Das K. C. Garcia M. Förster B. Zech W. (2008). Charcoal and smoke extract stimulate the soil microbial community in a highly weathered xanthic Ferralsol. Pedobiologia 51, 359–366. doi: 10.1016/j.pedobi.2007.08.002

[ref58] Sun X. Han X. Ping F. Zhang L. Zhang K. Chen M. (2018). Effect of rice-straw biochar on nitrous oxide emissions from paddy soils under elevated CO_2_ and temperature. Sci. Total Environ. 628-629, 1009–1016. 30045525 10.1016/j.scitotenv.2018.02.046

[ref59] Tang B. Xu H. Song F. Ge H. Chen L. Yue S. (2022). Effect of biochar on immobilization remediation of cd rectanglecontaminated soil and environmental quality. Environ. Res. 204:111840. doi: 10.1016/j.envres.2021.111840, PMID: 34403669

[ref60] Tian J. Kuang X. Tang M. Chen X. Huang F. Cai Y. (2021). Biochar application under low phosphorus input promotes soil organic phosphorus mineralization by shifting bacterial phoD gene community composition. Sci. Total Environ. 779:146556. doi: 10.1016/j.scitotenv.2021.146556 34030240

[ref61] Ullah S. Liang H. Ali I. Zhao Q. Iqbal A. Wei S. (2020). Biochar coupled with contrasting nitrogen sources mediated changes in carbon and nitrogen pools, microbial and enzymatic activity in paddy soil. J. Saudi Chem. Soc. 24, 835–849. doi: 10.1016/j.jscs.2020.08.008

[ref62] Wang L. Gao C. Yang K. Sheng Y. Xu J. Zhao Y. (2021). Effects of biochar aging in the soil on its mechanical property and performance for soil CO_2_ and N_2_O emissions. Sci. Total Environ. 782:146824. doi: 10.1016/j.scitotenv.2021.146824 33839651

[ref63] Wang X. Li W. Xiao Y. Cheng A. Shen T. Zhu M. (2021). Abundance and diversity of carbon-fixing bacterial communities in karst wetland soil ecosystems. Catena 204:105418. doi: 10.1016/j.catena.2021.105418

[ref64] Wang H. Ren T. Muller K. Van Zwieten L. Wang H. Feng H. (2021). Soil type regulates carbon and nitrogen stoichiometry and mineralization following biochar or nitrogen addition. Sci. Total Environ. 753:141645. doi: 10.1016/j.scitotenv.2020.141645 33207475

[ref65] Wu J. Li Z. Huang D. Liu X. Tang C. Parikh S. J. (2020). A novel calcium-based magnetic biochar is effective in stabilization of arsenic and cadmium co-contamination in aerobic soils. J. Hazard. Mater. 387:122010. doi: 10.1016/j.jhazmat.2019.122010, PMID: 31927353

[ref66] Wu L. Wei C. Zhang S. Wang Y. Kuzyakov Y. Ding X. (2019). MgO-modified biochar increases phosphate retention and rice yields in saline-alkaline soil. J. Clean. Prod. 235, 901–909. doi: 10.1016/j.jclepro.2019.07.043

[ref67] Wu S. Zhuang G. Bai Z. Cen Y. Xu S. Sun H. (2018). Mitigation of nitrous oxide emissions from acidic soils by bacillus amyloliquefaciens, a plant growth-promoting bacterium. Glob. Chang. Biol. 24, 2352–2365. doi: 10.1111/gcb.14025, PMID: 29251817

[ref68] Xie G. X. Huang Q. T. Yang S. E. Qin Z. L. Liu L. H. Deng T. H. (2021). Extraction of citrus planting plots based on medium-high different images. J. Southern Agric. 52, 3454–3462.

[ref69] Xu H. Shao H. Lu Y. (2019). Arbuscular mycorrhiza fungi and related soil microbial activity drive carbon mineralization in the maize rhizosphere. Ecotoxicol. Environ. Saf. 182:109476. doi: 10.1016/j.ecoenv.2019.109476, PMID: 31352211

[ref70] Yadav V. Jain S. Mishra P. Khare P. Shukla A. K. Karak T. (2019). Amelioration in nutrient mineralization and microbial activities of sandy loam soil by short term field aged biochar. Appl. Soil Ecol. 138, 144–155. doi: 10.1016/j.apsoil.2019.01.012

[ref71] Yang X. Liu J. McGrouther K. Huang H. Lu K. Guo X. (2016). Effect of biochar on the extractability of heavy metals (cd, cu, pb, and Zn) and enzyme activity in soil. Environ. Sci. Pollut. Res. Int. 23, 974–984. doi: 10.1007/s11356-015-4233-0, PMID: 25772863

[ref72] Yang C. Lu S. (2022). Straw and straw biochar differently affect phosphorus availability, enzyme activity and microbial functional genes in an Ultisol. Sci. Total Environ. 805:150325. doi: 10.1016/j.scitotenv.2021.150325 34537703

[ref73] Yang X. Wang D. Lan Y. Meng J. Jiang L. Sun Q. (2017). Labile organic carbon fractions and carbon pool management index in a 3-year field study with biochar amendment. J. Soils Sediments 18, 1569–1578. doi: 10.1007/s11368-017-1874-2

[ref74] Yao T. Zhang W. Gulaqa A. Cui Y. Zhou Y. Weng W. (2021). Effects of Peanut Shell biochar on soil nutrients, soil enzyme activity, and Rice yield in heavily saline-sodic Paddy field. J. Soil Sci. Plant Nutr. 21, 655–664. doi: 10.1007/s42729-020-00390-z

[ref75] Ye R. Horwath W. R. (2017). Influence of rice straw on priming of soil C for dissolved organic C and CH_4_ production. Plant Soil 417, 231–241. doi: 10.1007/s11104-017-3254-5

[ref76] Yin Q. Wang R. Zhao Z. (2018). Application of mg–Al-modified biochar for simultaneous removal of ammonium, nitrate, and phosphate from eutrophic water. J. Clean. Prod. 176, 230–240. doi: 10.1016/j.jclepro.2017.12.117

[ref77] Zhang M. Gao B. Yao Y. Xue Y. Inyang M. (2012). Synthesis of porous MgO-biochar nanocomposites for removal of phosphate and nitrate from aqueous solutions. Chem. Eng. J. 210, 26–32. doi: 10.1016/j.cej.2012.08.052

[ref78] Zhang Y. X. Li D. Zhang Z. Y. Liao K. J. (2010). A comparison study of two methods for mensuration of soil cation exchange capacity. Guizhou Forestry Sci. Technol. 38, 45–49.

[ref79] Zhang X. Teng Z. Zhang H. Cai D. Zhang J. Meng F. (2021). Nitrogen application and intercropping change microbial community diversity and physicochemical characteristics in mulberry and alfalfa rhizosphere soil. J. For. Res. 32, 2121–2133. doi: 10.1007/s11676-020-01271-y

[ref80] Zhang H. Ullah F. Ahmad R. Ali Shah S. U. Khan A. Adnan M. (2022). Response of soil proteobacteria to biochar amendment in sustainable agriculture—a mini review. J. Soil Plant Environ. 1, 16–30. doi: 10.56946/jspae.v1i2.56

[ref81] Zhang Z. Wang W. Qi J. Zhang H. Tao F. Zhang R. (2018). Priming effects of soil organic matter decomposition with addition of different carbon substrates. J. Soils Sediments 19, 1171–1178. doi: 10.1007/s11368-018-2103-3

[ref82] Zhang Q. Xiao J. Xue J. Zhang L. (2020). Quantifying the effects of biochar application on greenhouse gas emissions from agricultural soils: a global meta-analysis. Sustainability 12:3436. doi: 10.3390/su12083436

[ref83] Zhang T. Zhu X. Shi L. Li J. Li S. Lu J. (2017). Efficient removal of lead from solution by celery-derived biochars rich in alkaline minerals. Bioresour. Technol. 235, 185–192. doi: 10.1016/j.biortech.2017.03.109, PMID: 28365346

[ref84] Zhao Z. Zhang C. Li F. Gao S. Zhang J. (2020). Effect of compost and inorganic fertilizer on organic carbon and activities of carbon cycle enzymes in aggregates of an intensively cultivated vertisol. PLoS One 15:e0229644. doi: 10.1371/journal.pone.0241371, PMID: 32163434 PMC7067440

