# Supplementary material for: Effects of magnesium-modified biochar on soil organic carbon mineralization in citrus orchard
Source: Front Microbiol. 2023 Jan 27;14:1109272. doi: 10.3389/fmicb.2023.1109272 (PMC9911438; doi:10.3389/fmicb.2023.1109272)
Supplement: Supplementary file 1 [file Data_Sheet_1.PDF]

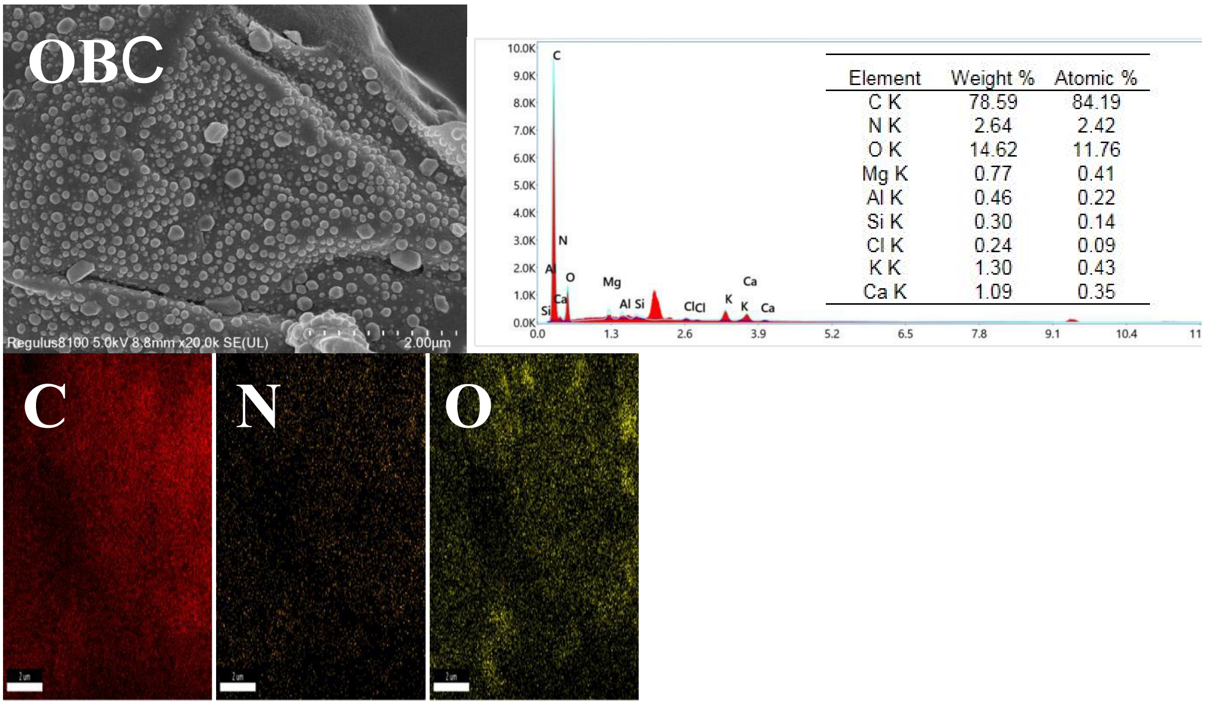

a

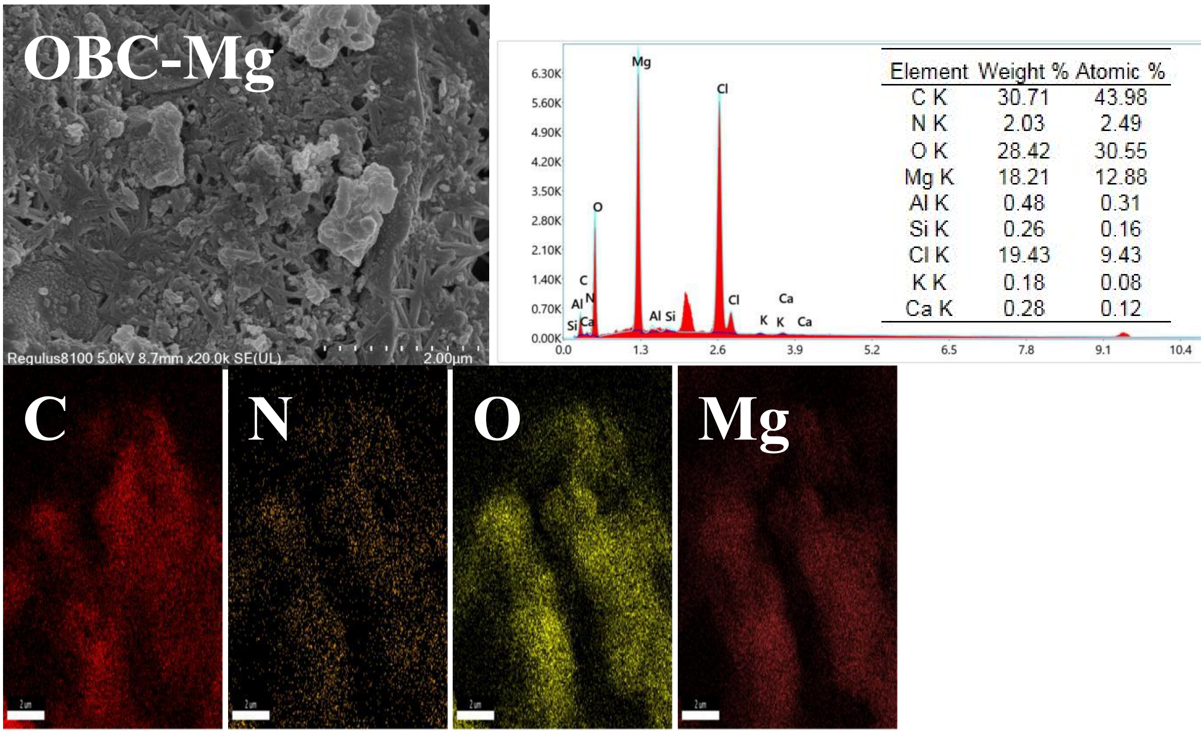

b

Fig. 1. SEM-EDS analysis of a) OBC and b) OBC-Mg

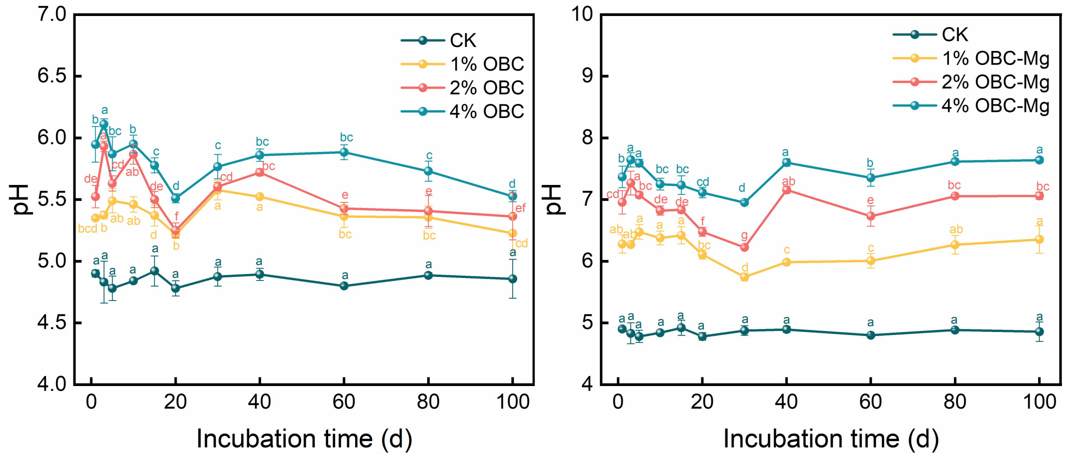

Fig. 2. Changes in soil pH after the amendment of OBC and OBC-Mg

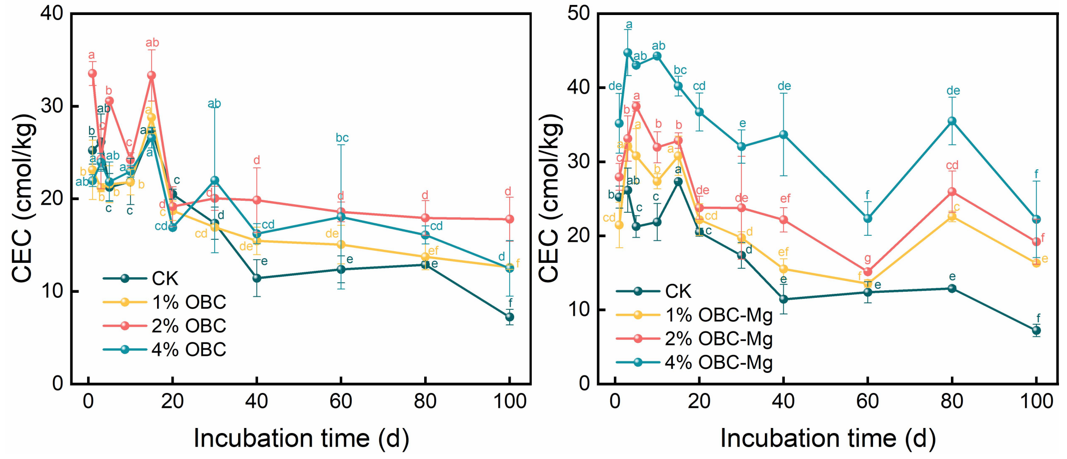

Fig. 3. Changes in soil CEC (cation exchange capacity) after the amendment of OBC and OBC-Mg

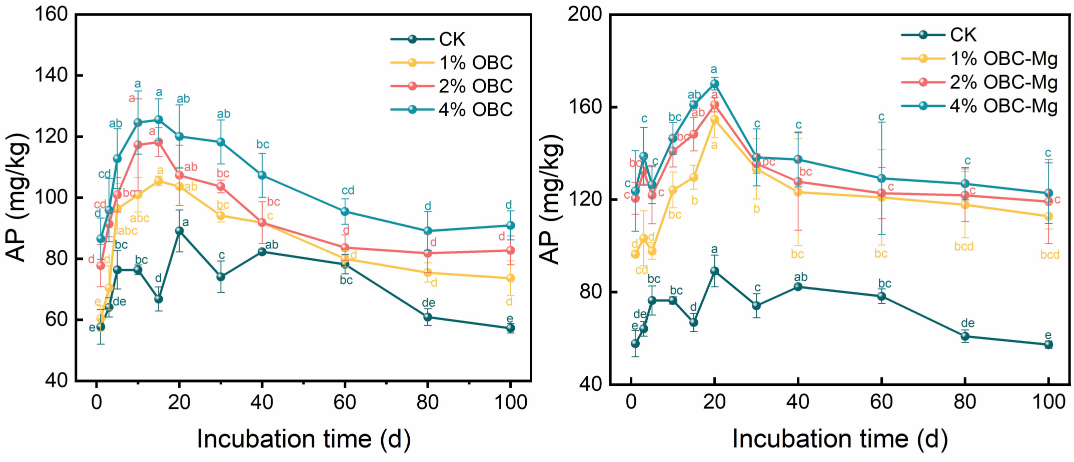

Fig. 4. Changes in soil AP (available phosphorus) after the amendment of OBC and OBC-Mg

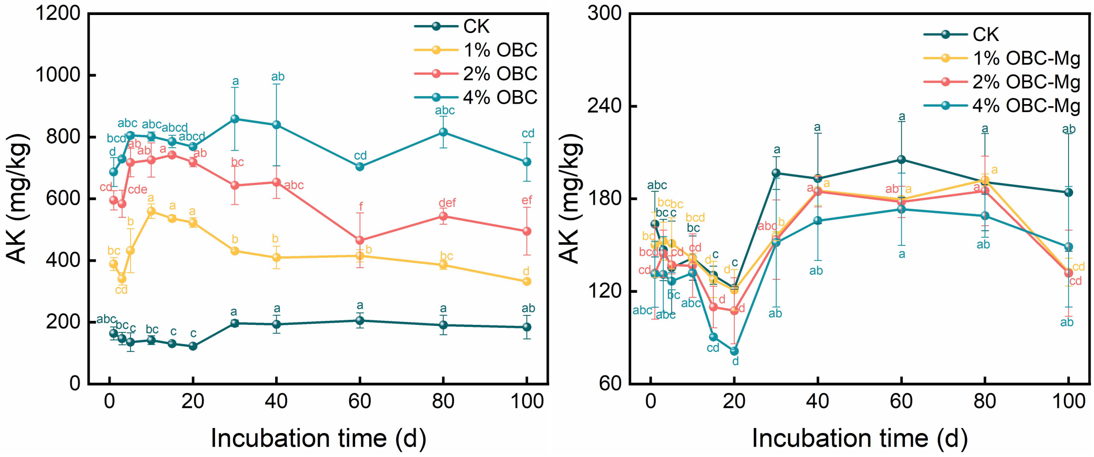

Fig. 5. Changes in soil AK (available potassium) after the amendment of OBC and OBC-Mg

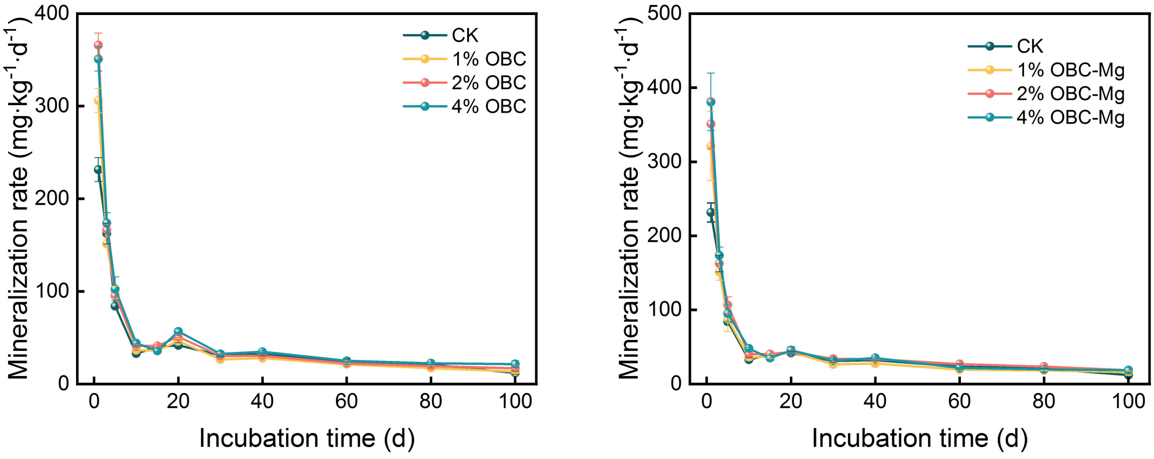

Fig. 6. Changes in soil mineralization rate after the amendment of OBC and OBC-Mg

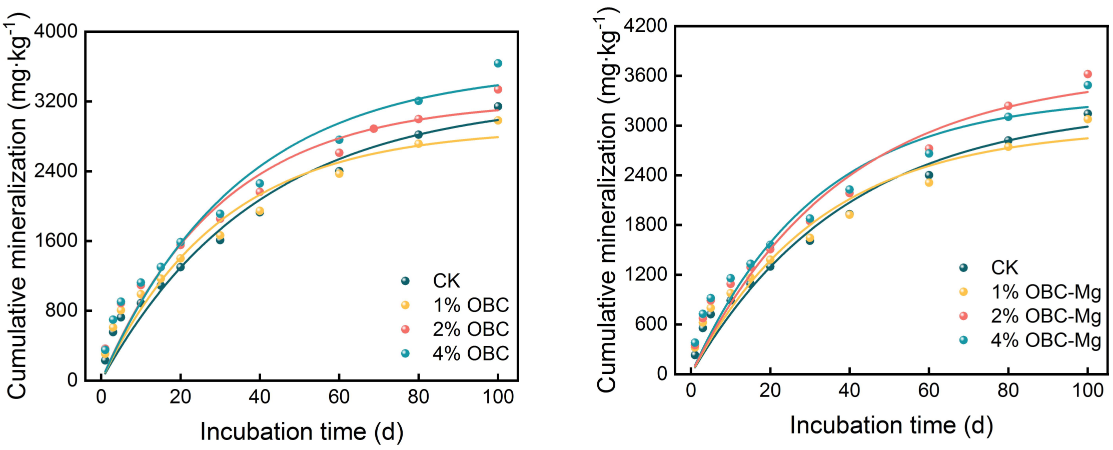

Fig. 7. Changes in soil cumulative mineralization after the amendment of OBC and OBC-Mg

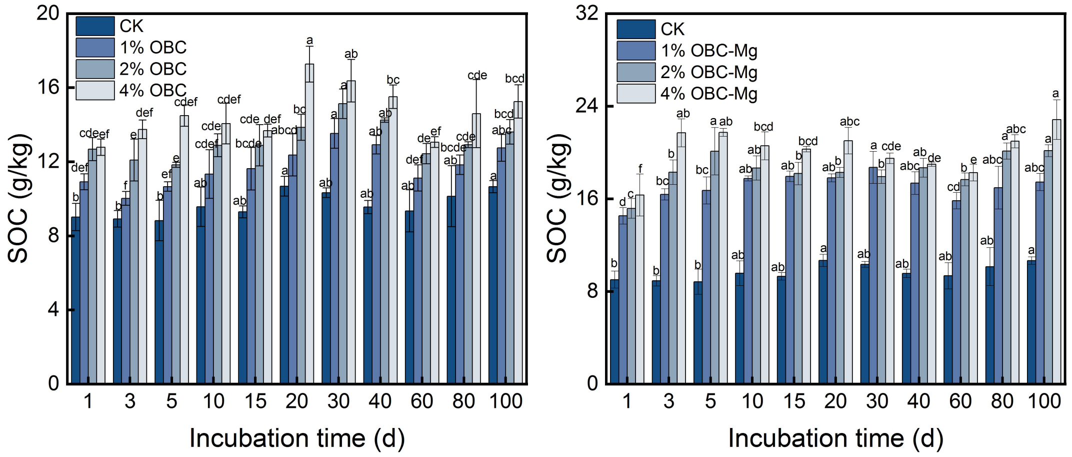

Fig. 8. Changes in SOC (soil organic carbon) after the amendment of OBC and OBC-Mg

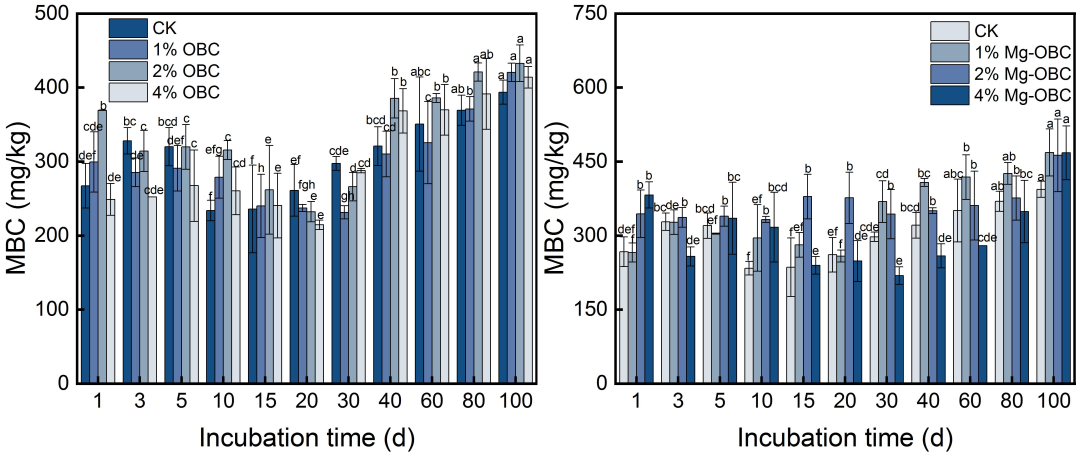

Fig. 9. Changes in MBC (microbial biomass carbon) after the amendment of OBC and OBC-Mg

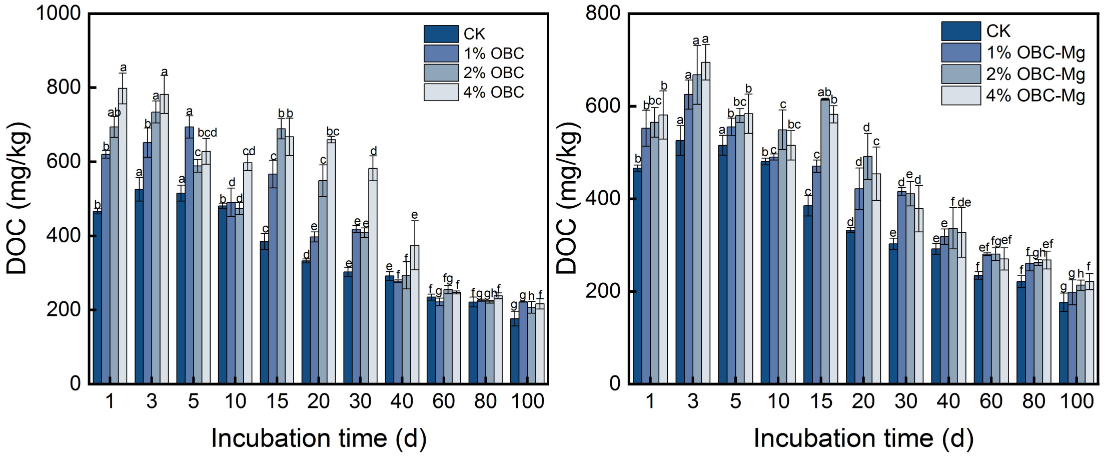

Fig. 10. Changes in DOC (dissolved organic carbon) after the amendment of OBC and OBC-Mg

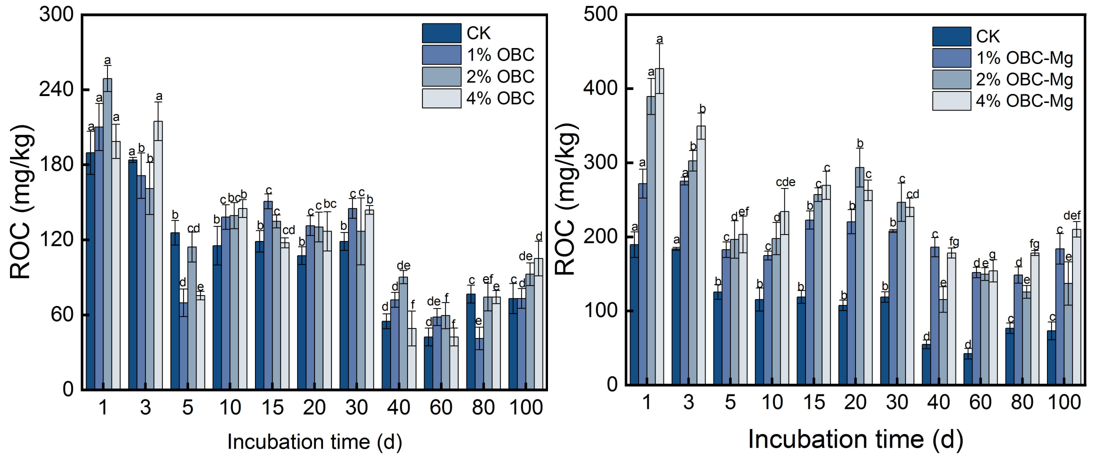

Fig. 11. Changes in ROC (readily oxidized organic carbon) after the amendment of OBC and OBC-Mg

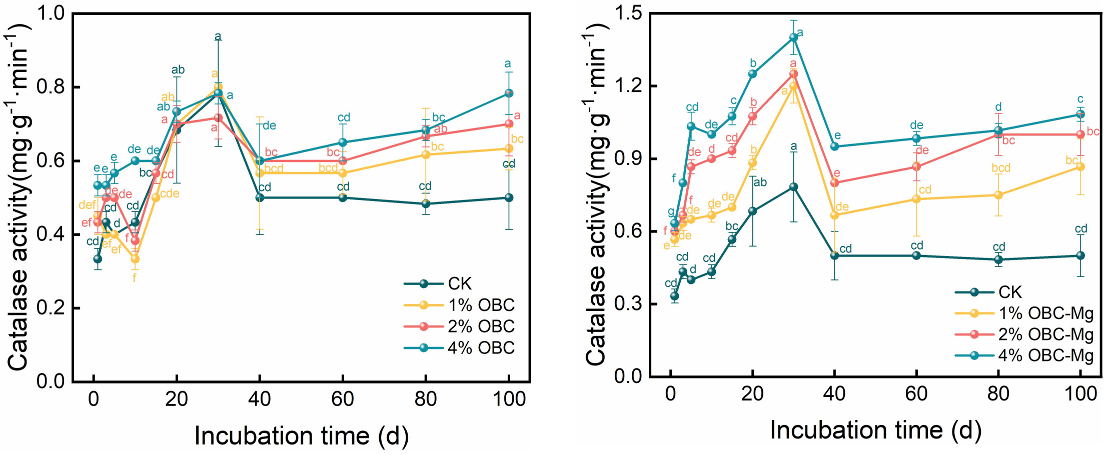

Fig. 12. Changes in soil catalase activity after the amendment of OBC and OBC-Mg

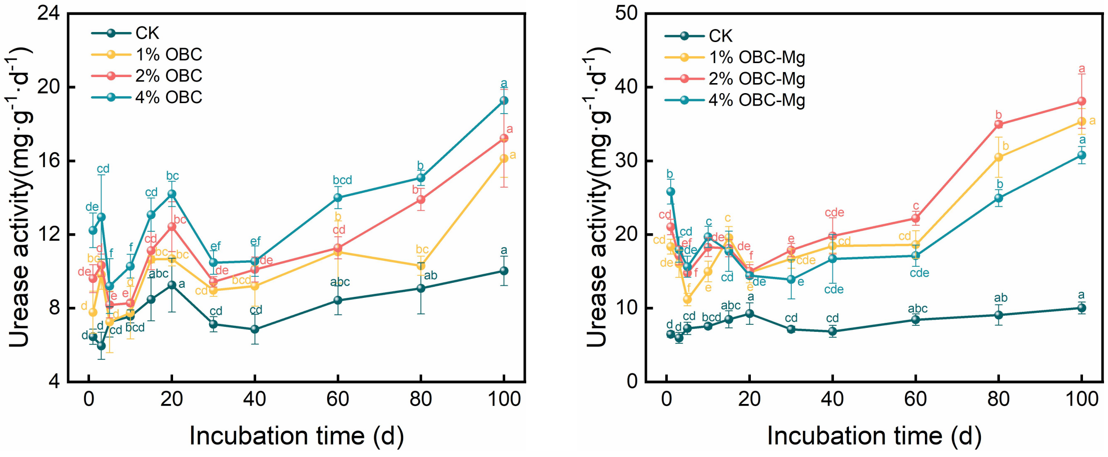

Fig. 13. Changes in soil urease activity after the amendment of OBC and OBC-Mg

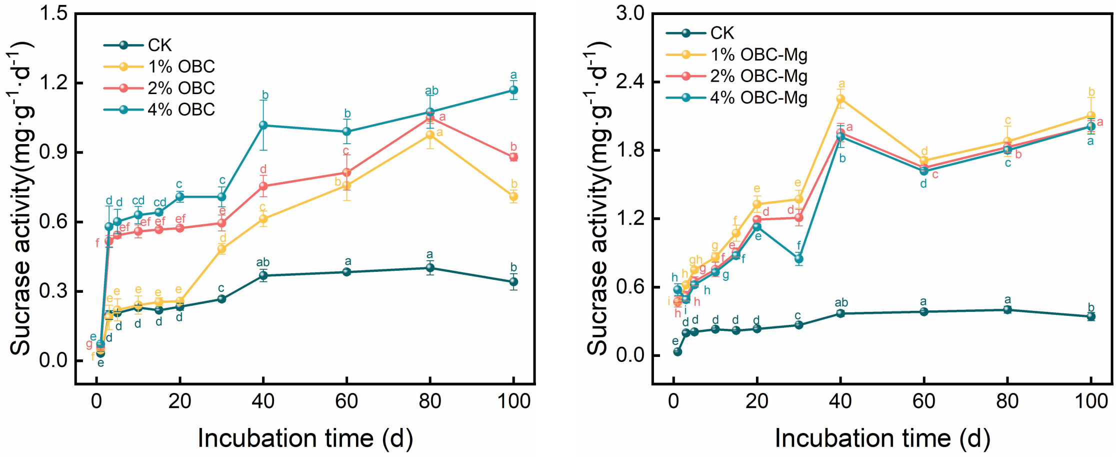

Fig. 14. Changes in soil sucrase activity after the amendment of OBC and OBC-Mg

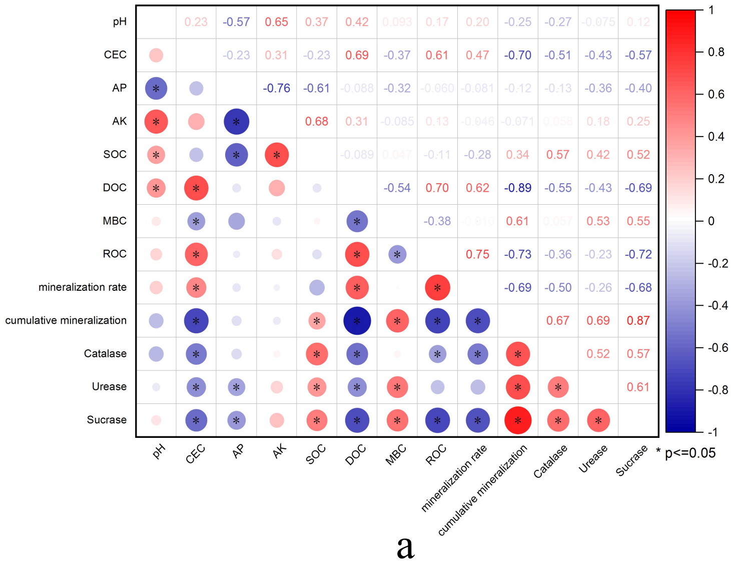

a

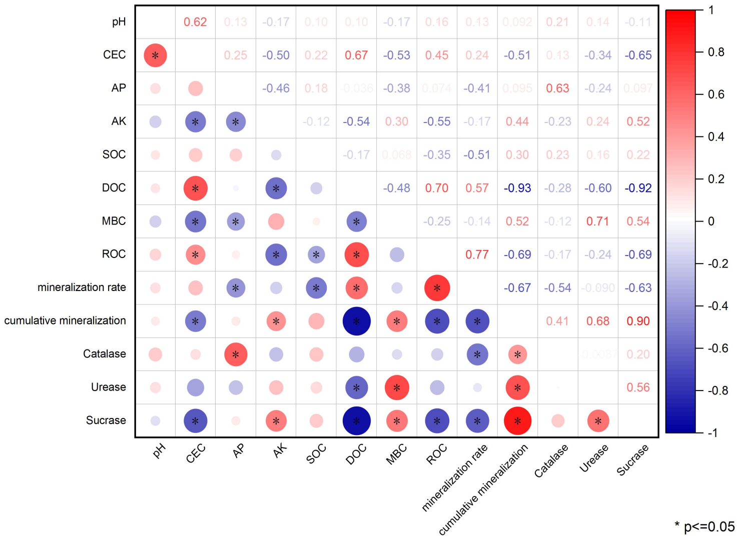

b

Figure 15. Correlation between physical and chemical properties, carbon composition, enzyme activity and mineralization of a) OBC-Mg and b) OBC-Mg
