# Supplementary material for: Effects of magnesium-modified biochar on soil organic carbon mineralization in citrus orchard
Source: Front Microbiol. 2023 Jan 27;14:1109272. doi: 10.3389/fmicb.2023.1109272 (PMC9911438; doi:10.3389/fmicb.2023.1109272)
Supplement: Supplementary file 2 [file Data_Sheet_2.docx]

**Supplementary Table 1. ANOVA/statistical indicators of CK**

| **Indicators** | **Average Square** | **F** |
| --- | --- | --- |
| **pH** | 0.007 | 1.142 |
| **CEC** | 135.534 | 100.305 |
| **AP** | 331.937 | 18.783 |
| **AK** | 2773.926 | 5.077 |
| **Mineralization rate** | 14534.394 | 488.433 |
| **SOC** | 5.543 | 1.965 |
| **MBC** | 8486.001 | 8.438 |
| **DOC** | 46602.769 | 175.07 |
| **ROC** | 6697.057 | 67.288 |
| **Catalase activity** | 0.04 | 6.663 |

| **Urease activity** | 4.933 | 6.026 |
| --- | --- | --- |
| **Sucrase activity** | 0.035 | 95.379 |

Note: CEC: cation exchange capacity; AP: available phosphorus; AK: available potassium; SOC: soil organic carbon; MBC: microbial biomass carbon; DOC: dissolved organic carbon; ROC: readily oxidized organic carbon.

**Supplementary Table 2. ANOVA/statistical indicators of OBC**

|  | **1% OBC** |  | **2% OBC** |  | **4% OBC** |  |
| --- | --- | --- | --- | --- | --- | --- |
| **Indicators** |  |  |  |  |  |  |
|  | **Average Square** | **F** | **Average Square** | **F** | **Average Square** | **F** |
| **pH** | 0.038 | 6.826 | 0.133 | 0.096 | 0.096 | 13.765 |
| **CEC** | 69.945 | 52.884 | 119.369 | 42.402 | 54.137 | 8.396 |
| **AP** | 694.16 | 24.442 | 620.342 | 11.505 | 666.988 | 10.57 |
| **AK** | 17566.249 | 21.929 | 28185.062 | 10.499 | 9795.102 | 2.863 |
| **Mineralization rate** | 23412.631 | 1128.068 | 32738.949 | 1488.167 | 30150.718 | 662.231 |

| **SOC** | 13.632 | 5.415 | 11.539 | 6.099 | 22.901 | 6.994 |
| --- | --- | --- | --- | --- | --- | --- |
| **MBC** | 11363.623 | 24.58 | 15346.313 | 52.552 | 15057.251 | 30.664 |
| **DOC** | 98871.768 | 185.682 | 120025.21 | 199.485 | 142543.873 | 113.078 |
| **ROC** | 8858.732 | 75.522 | 7872.266 | 42.837 | 9511.058 | 85.487 |
| **Catalase activity** | 0.06 | 11.197 | 0.039 | 25.53 | 0.025 | 13.408 |
| **Urease activity** | 17.644 | 15.61 | 21.08 | 18.278 | 24.192 | 21.891 |
| **Sucrase activity** | 0.26 | 165.554 | 0.194 | 182.339 | 0.285 | 87.625 |

Note: CEC: cation exchange capacity; AP: available phosphorus; AK: available potassium; SOC: soil organic carbon; MBC: microbial biomass carbon; DOC: dissolved organic carbon; ROC: readily oxidized organic carbon.

**Supplementary Table 3. ANOVA/statistical indicators of OBC-Mg**

**Indicators**

**1% OBC-Mg 2% OBC-Mg 4% OBC-Mg**

**Average Square F Average Square F Average Square F**

| **pH** | 0.148 | 9.867 | 0.286 | 25.006 | 0.173 | 16.642 |
| --- | --- | --- | --- | --- | --- | --- |
| **CEC** | 127.9 | 57.933 | 135.594 | 32.387 | 182.137 | 31.071 |
| **AP** | 864.945 | 5.701 | 541.217 | 4.312 | 721.07 | 4.7 |
| **AK** | 1662.516 | 13.071 | 2237.748 | 5.624 | 2695.749 | 5.133 |
| **Mineralization rate** | 25410.046 | 106.563 | 29829.571 | 245.916 | 35638.614 | 216.364 |
| **SOC** | 15.886 | 4.466 | 24.399 | 6.499 | 40.475 | 9.529 |
| **MBC** | 16184.72 | 20.819 | 4083.811 | 2.122 | 16581.997 | 10.872 |
| **DOC** | 56949.814 | 100.095 | 75165.54 | 67.519 | 76274.888 | 52.223 |
| **ROC** | 5454.67 | 33.506 | 22713.63 | 53.969 | 19566.765 | 52.276 |
| **Catalase activity** | 0.092 | 13.832 | 0.099 | 50.841 | 0.123 | 137.868 |
| **Urease activity** | 151.846 | 59.358 | 180.244 | 70.277 | 86.332 | 23.677 |
| **Sucrase activity** | 1.129 | 179.743 | 1.021 | 401.28 | 1.008 | 461.392 |

Note: CEC: cation exchange capacity; AP: available phosphorus; AK: available potassium; SOC: soil organic carbon; MBC: microbial biomass carbon; DOC: dissolved organic carbon; ROC: readily oxidized organic carbon.
